# Isolation, Characterization and In Silico Studies of Secondary Metabolites from the Whole Plant of *Polygala inexpectata* Peşmen & Erik

**DOI:** 10.3390/molecules27030684

**Published:** 2022-01-21

**Authors:** Ayşe Ünlü, Kerem Teralı, Zübeyde Uğurlu Aydın, Ali A. Dönmez, Hasan Soliman Yusufoğlu, İhsan Çalış

**Affiliations:** 1 Department of Biology, Faculty of Science, Hacettepe University, Ankara 06800, Turkey; zubeydeugurlu@hacettepe.edu.tr (Z.U.A.); donmez@hacettepe.edu.tr (A.A.D.); 2 Department of Medical Biochemistry, Faculty of Medicine, Girne American University, Kyrenia 99428, Cyprus; keremterali@gau.edu.tr; 3 Department of Pharmacognosy & Pharmaceutical Chemistry, College of Dentistry & Pharmacy, Buraydah Private Colleges, Buraydah 51418, Saudi Arabia; yusufoglu@psau.edu.sa; 4 Department of Pharmacognosy, Faculty of Pharmacy, Near East University, Nicosia 99138, Cyprus; ihsan.calis@neu.edu.tr

**Keywords:** liriodendrin, molecular docking, ombuoside, sucrose esters, syringin

## Abstract

*Polygala* species are frequently used worldwide in the treatment of various diseases, such as inflammatory and autoimmune disorders as well as metabolic and neurodegenerative diseases, due to the large number of secondary metabolites they contain. The present study was performed on *Polygala inexpectata*, which is a narrow endemic species for the flora of Turkey, and resulted in the isolation of nine known compounds, 6,3′-disinapoyl-sucrose (**1**), 6-*O*-sinapoyl,3′-*O*-trimethoxy-cinnamoyl-sucrose (tenuifoliside C) (**2**), 3′-*O*-(*O*-methyl-feruloyl)-sucrose (**3**), 3′-*O*-(sinapoyl)-sucrose (**4**), 3′-*O*-trimethoxy-cinnamoyl-sucrose (glomeratose) (**5**), 3′-*O*-feruloyl-sucrose (sibiricose A5) (**6**), sinapyl alcohol 4-*O*-glucoside (syringin or eleutheroside B) (**7**), liriodendrin (**8**), and 7,4′-di-*O*-methylquercetin-3-*O*-β-rutinoside (ombuin 3-*O*-rutinoside or ombuoside) (**9**). The structures of the compounds were determined by the spectroscopic methods including 1D-NMR (^1^H NMR, ^13^C NMR, DEPT-135), 2D-NMR (COSY, NOESY, HSQC, HMBC), and HRMS. The isolated compounds were shown in an in silico setting to be accommodated well within the inhibitor-binding pockets of myeloperoxidase and inducible nitric oxide synthase and anchored mainly through hydrogen-bonding interactions and π-effects. It is therefore plausible to suggest that the previously established anti-inflammatory properties of some *Polygala*-derived phytochemicals may be due, in part, to the modulation of pro-inflammatory enzyme activities.

## 1. Introduction

*Polygala* L. is the largest genus of the family *Polygalaceae* with more than 700 recognized species and is distributed in all continental areas except the Arctic and New Zealand [1,2,3]. The genus is represented by 16 native and one cultivated species in Turkey [4,5,6,7]. Recently, two new species in the subgenus *Polygala* have been described from the Eastern part of Turkey [8,9]. Among *Polygala* taxa, *P. inexpectata* Peşmen & Erik, which is subject to this study, is a narrow endemic species and is known from only the type locality, Ermenek, Turkey.

The *Polygala* species represent a rich molecular diversity in terms of plant phytochemical constituents, and they have widely been used in folk medicine for a long time to treat chronic asthma, bronchial asthma, and whooping cough as an expectorant and stimulant in many countries such as China, Japan, Thailand, India, North-South America, Brazil, and Turkey [10,11,12,13,14,15,16]. For instance, the roots of *P. tenuifolia* Willd. and *P. senega* L. are the two species with the most important plant components in Traditional Chinese Medicine (TCM). Both are species included in the German (DAB), European (Ph. Eur.), Austrian (ÖAB), Indian Ayurvedic, British Herbal, Swiss (Ph. Helv.), Japanese Pharmacopoeias and ESCOP, Commission E, and WHO Monographs. Besides, more than 140 compounds have been isolated from the *Polygala* species [17]. These compounds are mostly xanthones [18,19,20,21,22,23,24,25,26,27,28,29], saponins [30,31,32,33], and oligosaccharides [34,35,36,37,38,39,40,41]. Some other secondary metabolites include coumarins [42,43,44], flavonoids [45,46,47,48,49,50,51], sterols [52], and lignans [53,54,55,56,57,58]. Many pharmacological studies have revealed the important pharmacological properties of the roots of *Polygala tenuifolia* [59]. Its effects on the cardiovascular and nervous systems [60,61,62,63] are well known. It has also been reported to have adjuvant [64,65], anti-inflammatory [66,67], antifungal [68], antitumor [69], antinociceptive [70], and pain-reducing [71] effects. Last but not least, various studies have demonstrated that it has anxiolytic and sedative-hypnotic [72] and analgesic [73] effects.

Both cell culture and mouse models of inflammatory disease have demonstrated that *Polygala* crude extracts and pure compounds are likely to exert their anti-inflammatory effects by blocking major inflammation-related signaling pathways, reducing pro-inflammatory mediators and/or down regulating the expression of inducible enzymes [59]. However, the direct inhibitory effects of *Polygala* secondary metabolites on pro-inflammatory enzyme systems have not been evaluated before.

The main aim of this study was to isolate and structurally elucidate the secondary metabolites from *P. inexpectata* and predict the inhibitory activities of the isolated phytochemicals on pro-inflammatory enzyme systems using computational methods. Accordingly, nine compounds (**1**–**9**) were isolated and subsequently characterized through NMR and HRMS interpretations. Each of the identified compounds was then docked onto myeloperoxidase (MPO), cyclooxygenase-2 (COX-2), and inducible nitric oxide synthase (iNOS) in an in-silico setting.

## 2. Results

**Figure 1:**
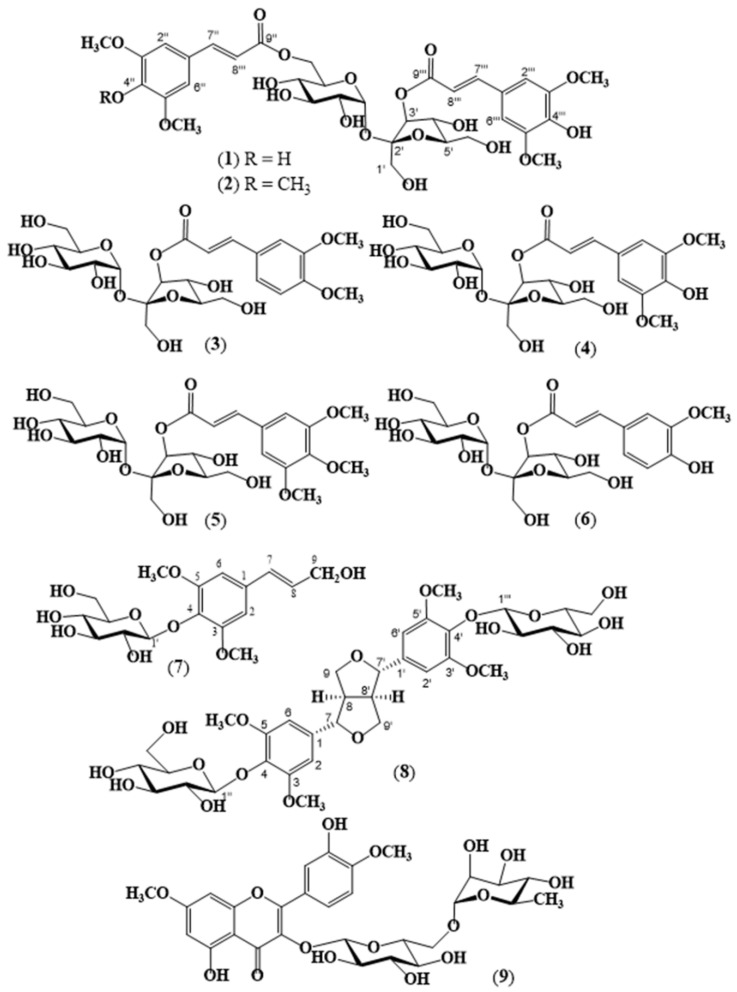
Chemical structures of compounds **1**–**9**: 6,3′-disinapoyl-sucrose (**1**)**,** 6-*O*-sinapoyl,3′-*O*-trimethoxy-cinnamoyl-sucrose (tenuifoliside C) (**2**), 3′-*O*-(*O*-methyl-feruloyl)-sucrose (**3**)**,** 3′-*O*-sinapoyl-sucrose (**4**), 3′-*O*-trimethoxy-cinnamoyl-sucrose (glomeratose) (**5**), 3′-*O*-feruloyl-sucrose (sibiricose A5) (**6**), sinapyl alcohol 4-*O*-glucoside (syringin or eleutheroside B) (**7**), liriodendrin (**8**), and a flavonol glycoside, 7,4-di-*O*-methylquercetin-3-*O*-β-rutinoside (ombuin 3-*O*-rutinoside or ombuoside) (**9**).

The aerial parts of *P. inexpectata* (150 g) were extracted using methanol (MeOH). After removing its lipophilic constituents using differential extraction in water (H_2_O) and dichloromethane (DCM), successive chromatographic techniques were applied for the fractionation and isolation of the secondary metabolites, as detailed in Section 4. In total, nine compounds were isolated, including six sucrose esters, namely 6,3′-disinapoyl-sucrose (**1**) [74], 6-*O*-sinapoyl,3′-*O*-trimethoxy-cinnamoyl-sucrose (tenuifoliside C) (**2**) [51,75], 3′-*O*-(*O*-methyl-feruloyl)-sucrose (**3**) [76], 3′-*O*-sinapoyl-sucrose (**4**) [77], 3′-*O*-trimethoxy-cinnamoyl-sucrose (glomeratose) (**5**) [78], and 3′-*O*-feruloyl-sucrose (sibiricose A5) (**6**) [79]. Additionally, a monomeric phenylpropane glycoside, sinapyl alcohol 4-*O*-glucoside (syringin or eleutheroside B) (**7**) [80,81], a tetrahydrofurofuran-type lignan diglycoside, liriodendrin (**8**) [82,83,84], and a flavonol glycoside, and 7,4-di-*O*-methylquercetin-3-*O*-β-rutinoside (ombuin 3-*O*-rutinoside or ombuoside) (**9**) [47,85] were isolated (Figure 1). The structure elucidation of the compounds **1**–**9** was based on the 1D- and 2D-NMR experiments (^1^H-, ^13^C-NMR, COSY, HSQC, HMBC, and NOESY) and were confirmed by the HRMS analysis (see Supplementary Materials). The ^1^H- and ^13^C-NMR spectral data of compounds **1**–**6** were presented in Table 1 and Table 2, respectively. Compounds **4** and **6** have been isolated as a mixture of other sucrose esters. The ^1^H- and ^13^C-NMR data based on 1D and 2D-NMR measurements as well as HRMS supported the proposed structures of **4** and **6**. The corresponding spectroscopic data presented in the experimental as well as in Table 1 and Table 2 were in good accordance with those reported [47,51,74,75,76,77,78,79,80,81,82,83,84,85].

**Figure 2:**
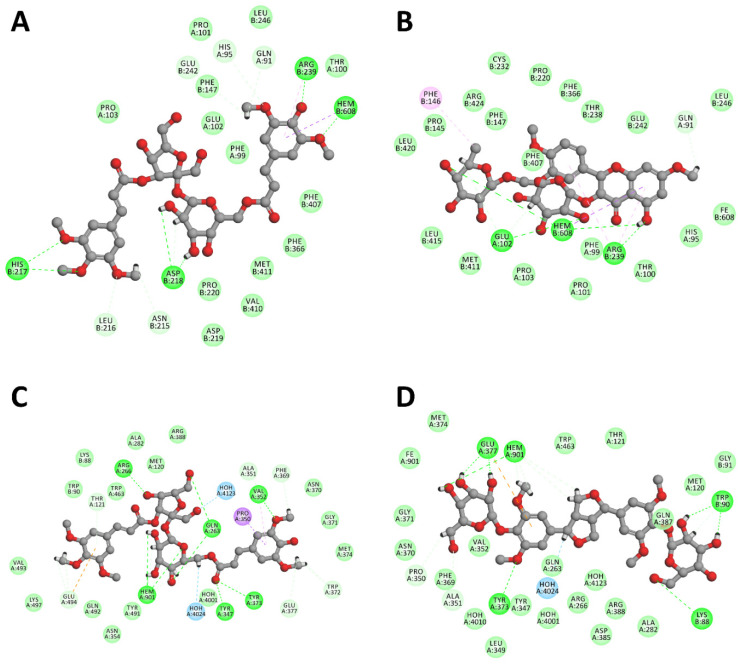

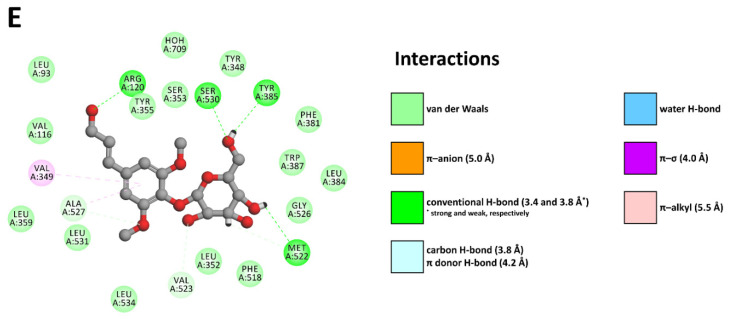
Results of cross-docking calculations showing (**A**) the top-ranking predicted binding pose of compound **2** within the active-site cleft of MPO, (**B**) the top-ranking predicted binding pose of compound **9** within the active-site cleft of MPO, (**C**) the top-ranking predicted binding pose of compound **2** within the active-site cleft of iNOS, (**D**) the top-ranking predicted binding pose of compound **8** within the active-site cleft of iNOS, and (**E**) the top-ranking predicted binding pose of compound **7** in the coxib-binding pocket of COX-2. The images were rendered using Discovery Studio Visualizer, v16.1.0 (Dassault Systèmes BIOVIA Corp., San Diego, CA, USA). A color-coding scheme was added to distinguish between the different types of non-covalent interactions, in which the maximum distance between two interacting centers was included in parentheses.

In an attempt to better understand the direct inhibitory effects of *P. inexpectata* secondary metabolites on pro-inflammatory enzyme systems, we docked nine compounds isolated from the plant on human counterparts of MPO, COX-2, and iNOS. The results of the redocking calculations revealed that JAMDA was able to reproduce the crystallographic binding modes of the *bona fide* enzyme inhibitors well, with root-mean-square deviations (RMSDs) of less than 1 Å (Table 3). In cross-docking experiments, all nine compounds tested were found to be able to occupy the inhibitor-binding pockets of MPO and iNOS, with similar or even higher docking scores compared to those of the cocrystallized inhibitors. The binding of the compounds in the inhibitor-binding pockets of MPO and iNOS appeared to be stabilized mainly by hydrogen-bonding interactions and π-effects (Figure 2). Two comparatively bulky sucrose esters (compounds **1** and **2**) in particular were predicted to be potent inhibitors of MPO. Besides forming favorable non-covalent interactions with key residues lining the active-site cleft of MPO (e.g., Gln91, His95, and Arg239), they were also able to interact with the enzyme’s heme prosthetic group through their sinapoyl moieties. Compound **9**, a flavonol glycoside, could also serve as a potent MPO inhibitor. Its best-scoring docking pose was estimated to engage in multiple non-covalent interactions with the heme. Compounds **1** and **2** were likely to inhibit iNOS as well. They could make interactions with invariant Glu377, other active-site residues involved in inhibitor binding (e.g., Gln263, Arg266, Tyr347), and the heme prosthetic group. Compound **8**, a tetrahydrofurofuran-type lignan diglycoside, emerged as a potentially superior inhibitor based on its docking score. It was able to form multiple non-covalent interactions with both Glu377 and the heme of iNOS through its pyranose and dimethoxybenzene rings. For COX-2, only compound **7**, a monomeric phenylpropane glycoside, was able to favorably bind to the enzyme, possibly due to its relatively small size. We do not, however, exclude the possibility that *Polygala*-derived phytochemicals may bind at an alternative site on COX-2 other than the coxib-binding site.

**Figure 3:**
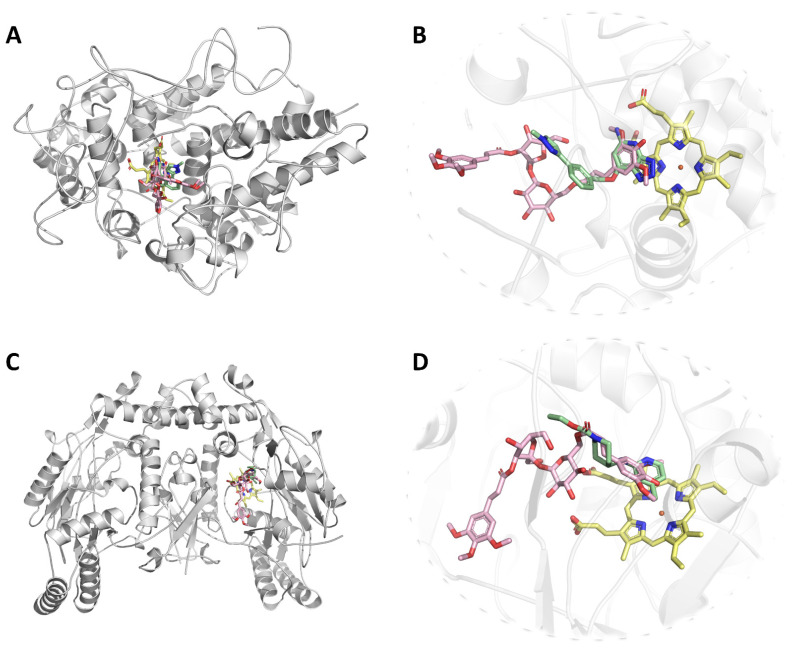
(**A**) Ribbon representation of the biological assembly of human MPO (PDB entry: 5QJ2), with docked compound **2** shown as pink sticks. (**B**) Close-up view of the superposed structures of compound **2** (pink) and the *bona fide* triazolopyridine-type inhibitor (green) in the active-site cavity of human MPO. The heme prosthetic group is shown as yellow sticks. (**C**) Ribbon representation of the biological assembly of human iNOS (PDB entry: 3E7G), with docked compound **2** shown as pink sticks. (**D**) Close-up view of the superposed structures of compound **2** (pink) and the *bona fide* aminopyridine-type inhibitor (green) in the active-site cavity of human iNOS. The heme prosthetic group is shown as yellow sticks. All images were rendered using the PyMOL Molecular Graphics System, v1.8 (Schrödinger LLC, Portland, OR, USA).

A glimpse at the superposed structures of docked compound **2** and the cocrystallized triazolopyridine compound revealed that the sinapoyl moiety of compound **2** and the heterocyclic core of the triazolopyridine compound stack on the heme prosthetic group of MPO, with their aromatic rings and polar functionalities (i.e., the oxygen atoms of sinapic acid and the nitrogen atoms of triazolopyridine) coinciding with each other (Figure 3). Similar correspondences in the positions of polar functionalities also exist between docked compound **2** and the *bona fide* iNOS inhibitor aminopyridine compound. These findings further validate the reliability of our molecular docking calculations.

## 3. Discussion

*P. inexpectata* is a narrow endemic taxon in Turkey. The phytochemistry of this species was studied in depth for the first time in this study. It is worth noting that most of the secondary metabolites isolated were sucrose ester derivatives. Additionally, a monomeric phenylpropane glycoside, a flavonol glycoside, and a tetrahydrofurofuran-type lignan diglycoside were also isolated from the whole plant of *P. inexpectata*. These sucrose esters, or, more specifically, phenylpropanoid sucrose esters, have a sucrose core linked to one or more Ph–CH=CH–CO– moieties (Ph: phenyl) via an ester bond. The ester-forming moieties can among others be substituted or unsubstituted sinapic, cinnamic, and ferulic acids, as observed in the present study. *Polygala* spp. are rich sources of phenylpropanoid sucrose esters in both mono- and disubstituted forms. Apart from the Polygalaceous plants, plant species of the Polygonaceae and Liliaceae families also represent major sources of sucrose mono- and diesters [86].

Accumulated data over the last decade suggest that the anti-inflammatory potential of the genus *Polygala* forms part of the basis for its widespread use in traditional medicine [17,59]. In a previous study, where the inhibitory activities of bioactive compounds from *P. tenuifolia* against lipopolysaccharide (LPS)-stimulated pro-inflammatory cytokine production in bone marrow-derived dendritic cells were tested, all isolated sucrose mono- and diesters were found to possess anti-inflammatory properties [33]. In another study on *P. japonica*, two sucrose esters, namely, tenuifolioside B and β-d-[3-*O*-(3,4,5-trimethoxycinnamoyl)]-fructofuranosyl-α-d-[6-*O*-(4-methoxybenzoyl)]-glucopyranoside, were shown to attenuate the release of pro-inflammatory cytokines in LPS-stimulated BV2 microglial cells [87]. These two studies provide direct evidence to support the notion that *Polygala* sucrose esters serve as natural products of significant therapeutic value, which can be exploited in anti-inflammatory therapy to particularly treat neuroinflammatory conditions. In fact, the important role played by sucrose esters in the fight against inflammation has been demonstrated not only for *Polygala*-derived phytochemicals but also for bioactive compounds extracted and purified from other plant species. For example, mono- and disubstituted sucrose esters from *Bidens parviflora* (Compositae) have been found to be effective in attenuating the release of histamine by rat mast cells stimulated by antigen–IgE antibody reaction and in suppressing the production of prostaglandin E_2_ by macrophages [88].

Our computational analyses reveal that secondary metabolites isolated from the whole plant of *P. inexpectata*, including phenylpropanoid sucrose esters with relatively high docking scores, may hold the potential to directly inhibit the heme-dependent pro-inflammatory enzymes iNOS and MPO. While intermittently increased inflammation is known to be required for survival during physical injury and infection, aberrant or chronic inflammation has been demonstrated to be associated with a number of diseases such as such as cardiovascular disease, cancer, diabetes mellitus, and autoimmune and neurodegenerative disorders [89]. iNOS is a normally silenced enzyme whose expression can be induced in a myriad of cells and tissues by certain cytokines and other pro-inflammatory factors. It catalyzes the production of nitric oxide (^·^NO) from l-arginine. ^·^NO is a gaseous free radical that, along with its oxidation products, can cause damage to biomolecules (lipids, DNA, and proteins) and tissues and induce necrosis or apoptosis [90]. MPO is an abundant enzyme expressed by activated immune cells of the myeloid lineage, particularly macrophages, monocytes, and neutrophils. It interacts with hydrogen peroxide (H_2_O_2_) to generate highly reactive species, such as hypochlorite (OCl^–^), superoxide (O_2_^·−^), and peroxynitrite (ONOO^–^), that can covalently modify lipids and thus lead to tissue injury [91]. MPO-generated free radicals can also induce apoptotic cell death and protein nitrotyrosination [92]. Furthermore, it has been shown that MPO increases the catalytic activity of iNOS by preventing ^·^NO feedback inhibition at sites of inflammation [93]. Therefore, both iNOS and MPO are engaged in a complex cascade of inflammatory events involving various cells and molecules. We believe that the previously established anti-inflammatory effects of *Polygala* extracts or components in animals and cell culture systems could be partly due to the modulation of pro-inflammatory enzyme activities.

## 4. Materials and Methods

### 4.1. General Experimental Procedures

Buchi^®^ rotavapor (R-210) with the heating bath (B-491), vacuum pump (V-700), and vacuum controller (V-850) was used for evaporation under low pressure. Christ^®^ Alpha 1–4 LD plus was used for the lyophilization of the samples. For medium pressure Liquid chromatography (MPLC), a Buchi Sepacore^®^ Chromatography system with Buchi borosilicate 3.3 columns (36 mm x 230 mm) packed with LiChroprep RP-18 (Merck, Darmstadt) was used. Reverse phase material LiChroprep C18 was used for vacuum liquid chromatography (VLC). Column chromatography was performed on silica gel 60 (0.063–0.200 mm; Merck, Darmstadt), and Sephadex™ LH-20 (GE Healthcare, Sweden) was also used for open column chromatography (CC) studies. Thin Layer Chromatography (TLC) analyses were performed on aluminum plates (Merck, Darmstadt) coated with silica gel 60 F254 and RP TLC. 1% Vanillin in MeOH and 5% H_2_SO_4_ relative in EtOH were used for spot detection on TLC plates. For NMR spectroscopy experiments, measurements were performed on a Bruker DRX 500 spectrometer operating at 500 MHz for ^1^H and 125 MHz for ^13^C, respectively.

### 4.2. Plant Material

The whole plant of *P. inexpectata* was collected from Karaman, around Ayrancı dam, steppe, 41°33′10.6″ N, 36°56′56.8″, 1265 m, May 2019, *A. A. Dönmez* 20373-*Z. Aydın*. The voucher specimen has been deposited at the Herbarium of the Faculty of Biology, Hacettepe University (HUB).

### 4.3. Extraction and Isolation

The air dried and powdered whole plants (leaves, stems, flowers, and roots) of P. inexpectata (150 g) were extracted two times with 95% MeOH by a refluxing process at 45 °C. The resulting extracts were combined, filtered, and concentrated under reduced pressure at 45 °C. The concentrated extract was diluted with water and partitioned with dichloromethane (DCM) to remove lipophilic compounds. The H_2_O phase was concentrated to 50 mL and subjected to column chromatography using polyamide G (Fluka) as a stationary phase and eluting with H_2_O and H_2_O–MeOH mixtures with an increasing amount of MeOH to afford six main fractions: A (16.70 g), B (596 mg), C (690 mg), D (314 mg), E (161 mg), and F (127 mg). Fraction A (16.70 g) was fractionated on a RP_VLC (Reversed Phase (LiChroprep C18, Vacuum Liquid Chromatography) column and eluted with increasing concentrations of MeOH in H_2_O (0%, 10%, 20%, and 100%) to obtain sixteen fractions (A1–A16). Fraction A was almost pure sucrose.

Fr. C (690 mg) was fractionated by MPLC with increasing concentrations of i-PrOH in H_2_O (up to 30%) to obtain nine subfractions (C1–C9). Fr. C3 was obtained as pure compound **1** (111 mg). Fr. C5 was obtained as pure compound **2** (51 mg).

Fr. B (596 mg) was fractionated by MPLC with increasing concentrations of i-PrOH in H_2_O (up to 30%) to obtain 11 subfractions (B1–B11). Fr. B3 was obtained as pure compound **1** (40 mg). Fr. B8 was obtained as pure compound **2** (54 mg). We found same result from NMR for chemical compound (**1**) 6,3′-disinapoyl-sucrose and compound (**2**) 6-*O*-sinapoyl-3′-*O*-trimethoxy-cinnamoyl-sucrose (tenuifoliside C).

Fr. A7 (360 mg) was subjected to a silica gel column using DCM-MeOH-H_2_O mixture (80:20:2→70:30:3) to afford compound (**3**) 3′-*O*-(*O*-methyl-feruloyl)-sucrose (41 mg).

Fr. A6 (277 mg) and Fr. A7_d_ was subjected to a silica gel column using DCM-MeOH-H_2_O mixture (80:20:2) to afford compounds (**7**) sinapyl alcohol 4-*O*-glucoside (syringin or eleutheroside B) (6 mg) and (**4**) 3′-*O*-(sinapoyl)-sucrose (114 mg).

Fr. A8 (342 mg) was subjected to a silica gel column using DCM-MeOH-H_2_O mixture (80:20:1→80:20:2) to afford compounds (**5**) 3′-*O*-trimethoxy-cinnamoyl-sucrose (glomeratose) (42 mg) and (**8**) liriodendrin (45 mg).

Fr. A13 (862 mg) was separated by gel chromatography (Sephadex LH-20), eluting with MeOH–H_2_O (1:1) to yield a total of six subfractions (Fr. A13a-f). Fr. A13c (70 mg) was purified by silica gel using DCM-MeOH-H_2_O (60:40:4) to obtain compound (**9**) 7,4′-di-*O*-methylquercetin-3-*O-*β-rutinoside (ombuin 3-*O*-rutinoside or ombuoside) (4 mg).

Fr. A5 (255 mg) was subjected to a silica gel column using DCM-MeOH-H_2_O mixture (80:20:1) to afford compound (**6**) 3′-*O*-feruloyl-sucrose (sibiricose A5) (16 mg).

Fr. A10 (531 mg) was rich in compound (**9**) 7,4′-di- *O*-methylquercetin-3-*O*-β-rutinoside (ombuin 3-*O*-rutinoside or ombuoside) and used for the re-isolation of **9**.

### 4.4. Physical and Spectral Data of Isolated Compounds 1 to 9

6,3′-disinapoyl-sucrose (**1**): Chemical formula C_34_H_42_O_19_; Mol. Wt. 754.6910; ^1^H-NMR (CD_3_OD, 500 MHz, δ [ppm]): Table 1; ^13^C-NMR (CD_3_OD, 125 MHz, δ [ppm]): Table 2; Positive ion HR-MS: *m*/*z* 777.2192 [M + Na]^+^; Negative ion HR-MS: *m*/*z* 753.2246 [M–H]^−^.

6-*O*-sinapoyl,3′-*O*-trimethoxy-cinnamoyl-sucrose (tenuifoliside C) (**2**): Chemical formula C_35_H_44_O_19_; Mol. Wt. 768.2477; ^1^H-NMR (CD_3_OD, 500 MHz, δ [ppm]): Table 1; ^13^C-NMR (CD_3_OD, 125 MHz, δ [ppm]): Table 2. Positive ion HR-MS: *m*/*z* 791.2362 [M + Na]^+^; Negative ion HR-MS: *m*/*z* 767.2393 [M–H]^−^.

3′-*O*-(*O*-methyl-feruloyl)-sucrose (**3**): Chemical formula C_23_H_32_O_14_; Mol. Wt. 532.4950; ^1^H-NMR (CD_3_OD, 500 MHz, δ [ppm]): Table 1; ^13^C-NMR (CD_3_OD, 125 MHz, δ [ppm]): Table 2; Positive ion HR-MS: *m*/*z* 555.1677 [M + Na]^+^; Negative ion HR-MS: *m*/*z* 531.1709 [M–H]^−^.

3′-*O*-(sinapoyl)-sucrose (**4**): Chemical formula C_23_H_32_O_15_; Mol. Wt. 548.4890: ^1^H-NMR (CD_3_OD, 500 MHz, δ [ppm]): Table 1; ^13^C-NMR (CD_3_OD, 125 MHz, δ [ppm]): Table 2; Positive ion HR-MS: *m*/*z* 571.1624 [M + Na]^+^; Negative ion HR-MS: *m*/*z* 547.1727 [M–H]^−^.

3′-*O*-trimethoxy-cinnamoyl-sucrose (glomeratose) (**5**): Chemical formula C_23_H_35_O_15_; Mol. Wt. 562.5210; ^1^H-NMR (CD_3_OD, 500 MHz, δ [ppm]): Table 1; ^13^C-NMR (CD_3_OD, 125 MHz, δ [ppm]): Table 2; Positive ion HR-MS: *m*/*z* 585.1781 [M + Na]^+^.

3′-*O*-feruloyl-sucrose (sibiricose A5) (**6**): Chemical formula C_22_H_30_O_14_; Mol. Wt. 518.4680; ^1^H-NMR (CD_3_OD, 500 MHz, δ [ppm]): Table 1; ^13^C-NMR (CD_3_OD, 125 MHz, δ [ppm]): Table 2; Positive ion HR-MS: *m*/*z* 541.1523 [M + Na]^+^; Negative ion HR-MS: *m*/*z* 517.1561 [M–H]^−^.

Sinapyl alcohol 4-*O*-glucoside (syringin or eleutheroside B) (**7**): Chemical formula C_17_H_24_O_9_; Mol. Wt. 372.1420; ^1^H-NMR (CD_3_OD, 500 MHz, δ [ppm]): Sinapyl alcohol moiety: 6.58 (s, 2H, H-2 and H-6); 6.50 (d, 1H, *J* = 11.9 Hz, H-7); 5.82 (dt, 1H, *J* = 11.9 and 6.5 Hz, H-8); 4.35 (dd, 2H, *J* = 1.1 and 6.5 Hz, H-9); 3.87 (s, 6H, 3/5-OMe); glucose moiety: 4.91 (overlapped, H-1′); 3.49 (dd, 1H, *J* = 7.8 and 9.0 Hz, H-2′); 3.50 (t, 1H, *J* = 9.0 Hz, H-3′); 3.45 (t, 1H, *J* = 9.0 Hz, H-4′); 3.45 (m, 1H, H-5′); 3.80 (dd, 1H, *J* = 12.0 and 2.3 Hz, H-6′a); 3.70 (dd, 1H, J = 12.0 and 5.3 Hz, H-6′).^13^C-NMR (CD_3_OD, 125 MHz, δ [ppm]): Sinapyl alcohol moiety: 134.73 (C-1); 107.96 (C-2/C-6); 153.97 (C-3/C-5); 134.44 (C-4); 132.50 (C-7); 131.46 (C-8); 59.78 (C-9); 57.06 (O*C*H_3_ × 2); glucose moiety: 105.22 (C-1′); 75.66 (C-2′); 77.56 (C-3′); 71.24 (C-4′); 78.29 (C-5′); 62.47 (C-6′). Positive ion HR-MS: *m*/*z* 395.1306 [M + Na]^+^.

Liriodendrin (**8**): Chemical formula C_34_H_46_O_14_; Mol. Wt. 742.7240; ^1^H-NMR (CD_3_OD, 500 MHz, δ [ppm]): Pinoresinol moiety: 6.67 (s, 4H, H-2, H-2′, H-6, H-6′); 4.66 (br s, 2H, H-7, H-7′); 3.09 (br s, H-8, H-8′); 4.21 (dd, 2H, H-9a, H-9′a); 3.84 (dd, 2H, H-9b, H-9′b); 3.77 (12 H, OC*H*_3_ × 4). Glucose moieties: 4.88 (d, 2H, *J* = 7.8 Hz, H-1″/H-1‴); 3.21 (m, 4 H, H-2″/H-2‴ and H-3″/H-3‴); 3.15 (m, 2H, H-4″/H-4‴); 3.05 (m, 2H, H-5″/H-5‴); 4.21 (dd, 2H, *J* = 11.4 and 1.8 Hz, H-6″a/H-6‴a); 3.42 (dd, 2 H, *J* = 11.4 and 6.0 Hz, H-6″b/H-6‴b). ^13^C-NMR (CD_3_OD, 125 MHz, δ [ppm]): Pinoresinol moiety: 137.60 (C-1/C-1′); 104.61 (C-2/C-2′ and C-6/C-6′); 153.08 (C-3/C-3′ and C-5/C-5′); 134.09 (C-4/C-4′); 85.53 (C-7/C-7′); 54.07 (C-8/C-8′); 71.83 (C-9/C-9′); 56.88 (O*C*H_3_ × 4). Glucose moieties: 103.11 (C-1″/C-1‴); 74.61 (C-2″/C-2‴); 76.94 (C-3″/C-3‴); 70.34 (C-4″/C-4‴); 77.63 (C-5″/C-5‴); 61.33 (C-6″/C-6‴). Positive ion HR-MS: *m*/*z* 765.2576 [M + Na]^+^.

Ombuin 3-*O*-rutinoside (ombuoside) [7,4-di-*O*-methylquercetin-3-*O*-β-rutinoside] (**9**): Chemical formula C_29_H_34_O_16_; Mol. Wt. 638.5750; ^1^H-NMR (DMSO-d_6_, 500 MHz, δ [ppm]): Flavonol moiety: 6.36 (br s, 1H, H-8); 6.67 (br s, 1H, H-6), 7.55 (br s, 1H, H-2′); 7.04 (d, *J* = 8.7 Hz, H-5′); 7.73 (br d, *J* = 8.7 Hz, H-6′); 3.85 (s, 3H, 7-OMe); 3.86 (s, 3H, 4′-OMe); 12.56 (s, 5-OH); glucose moiety: 5.39 (d, 1H, *J* = 7.0 Hz, H-1″); 3.26 (m, 1H, H-2″); 3.27 (m, 1H, H-3″); 3.51 (m, 1H, H-4″); 3.30 (m, 1H, H-5″); 3.72 (br d, 1H, *J* = 12.0 Hz, H-6″a); 3.35 (m, 1H, H-6″b); rhamnose moiety: 4.40 (br s, 1H, H-1‴); 3.41 (br d, 1H, *J* = 3.2 Hz, H-2‴); 3.10 (dd, 1H, *J* = 3.2 and 9.2 Hz, H-3‴); 3.08 (t 1H, J = 9.2 Hz, H-4‴); 3.29 (m, 1H, H-5‴); 0.97 (d, 3H, *J* = 5.8 Hz, H-6‴); ^13^C-NMR (DMSO-d_6_, 125 MHz, δ [ppm]): Flavonol moiety: 157.21 (C-2); 134.27 (C-3); 178.13 (C-4); 161.43 (C-5); 98.56 (C-6); 165.75 (C-7); 92.84 (C-8); 157.02 (C-9); 105.61 (C-10); 122.13 (C-1′); 116.38 (C-2′); 146.46 (C-3′); 150.63 (C-4′); 111.92 (C-5′); 123.00 (C-6′); 56.67 (7-OCH_3_); 56.21 (4′-OCH_3_); glucose moiety: 101.76 (C-1″); 74.63 (C-2″); 76.97 (C-3″); 70.42 (C-4″); 76.40 (C-5″); 67.49 (C-6″). rhamnose moiety: 101.38 (C-1‴); 70.94 (C-2‴); 71.17 (C-3‴); 72.39 (C-4‴); 68.84 (C-5‴); 18.32 (C-6‴); Positive ion HR-MS: *m*/*z* 639.1909 [M + H]^+^, *m*/*z* 661.1726 [M + Na]^+^; Negative ion HR-MS: *m*/*z* 637.1761 [M–H]^−^.

### 4.5. Protein-Ligand Docking

The energy-minimized 3D conformers of *P. inexpectata* secondary metabolites were generated based on the corresponding SMILES strings using myPresto programs available at https://demo1.biomodeling.co.jp/, accessed on 11 June 2021. The X-ray crystallographic structures of (i) human myeloperoxidase (MPO) in complex with a potent triazolopyridine compound [94], (ii) human cyclooxygenase-2 (COX-2) in complex with a selective inhibitor of the coxib type [95], and (iii) human inducible nitric oxide synthase (iNOS) in complex with a selective aminopyridine compound [96] were retrieved from the RCSB Protein Data Bank [97] available at https://www.rcsb.org/, accessed on 23 July 2021. These enzymes not only engage in inflammatory processes but also in the generation of free radicals and the oxidation of nucleic acids, lipids, and proteins (please see Section 3 for details). Ligand-bound (holo) conformational states of pro-inflammatory enzymes in complex with potent and selective inhibitors were selected rationally as target structures for docking. They were also chosen based on their acquisition technique (X-ray crystallography), resolution (less than 3 Å), and structure completeness. The structures were prepared using the Dock Prep utility of UCSF Chimera version 1.11.2 [98]. For atoms with alternate locations, only the highest-occupancy set was retained. Additionally, each truncated side chain was replaced with a complete side chain of the same residue type using the Dunbrack rotamer library. Missing hydrogen atoms were added to the structures using Protoss [99,100], available at https://proteins.plus/, accessed on 23 July 2021. The ligands were docked in the presence of cofactors and structurally relevant water molecules into the inhibitor-binding pockets of the proteins using JAMDA [101,102,103], available at https://proteins.plus/, accessed on 23 July 2021. Each docking site was defined by the cocrystallized inhibitor, with a site radius of 6.5 Å. Protein–ligand docking was executed with high precision.

## 5. Conclusions

Overall, the present study attempted to explore the phytochemical composition of the endemic taxon *P. inexpectata* from Turkey. The plant appears to be comparable to other *Polygala* species with medicinal uses in that it has a rich content of sucrose esters. Liriodendrin and ombuoside are the isolated lignin and flavone glycosides, respectively, and are normally rare in this genus. The findings of the molecular docking calculations highlight the obtained compounds’ potential to inhibit the pro-inflammatory enzymes iNOS and MPO. The very encouraging in silico results indicate that these phytochemicals may have a future role in anti-inflammatory drug development research. Further in vitro and/or in vivo studies, however, are required to demonstrate their efficacy. These data could prove valuable in extending the knowledge of the phytochemistry and medicinal properties of the *Polygala* taxa.

## Figures and Tables

**Table 1:** ^1^H-NMR data of sucrose esters (**1–6**) (δ_H_ 500 MHz, CD_3_OD).

	1	2	3	4	5	6
H–Atom	δ_H_, ppm	δ_H_, ppm	δ_H_, ppm	δ_H_, ppm	δ_H_, ppm	δ_H_, ppm
1	5.53 d (3.8)	5.52 d (3.8)	5.45 d (3.4)	5.45 d (3.5)	5.44 d (3.6)	5.46 d (3.6)
2	3.51 dd (3.8/9.7)	3.51 dd (3.8/9.7)	3.46 (3.4/9.8)	3.48 dd (3.5/9.5)	3.45 dd (3.6/9.5)	3.49 dd (3.6/9.5)
3	3.70 t (9.2)	3.69 t (9.2)	3.68 t (9.3)	3.68 t	3.67 t (9.5)	3.70 t (9.5)
4	3.34 t (9.3)	3.34 t (9.3)	3.43 t (9.3)	3.43 t (9.5)	3.42 t (9.5)	3.45 t (9.5)
5	4.29 gdd (9.3/7.3)	4.28 gdd (9.3/7.3)	3.92 m	3.92 m	3.92 m	3.94 m
6	4.23 dd (11.7/7.3) 4.69 gd (11.7)	4.23 dd (11.7/7.3) 4.69 gd (11.7)	3.84 † 3.78 dd (12.2/4.4)	3.85/3.79	3.84 dd (12.0/2.1) 3.78 dd (12.0/4.6)	3.86 † 3.81 †
1′	3.65 d (12.2)	3.65 d (12.2)	3.68 d (12.3)	3.64 d (12.1)	3.66 d (12.2)	3.69 d (12.2)
	3.61 d (12.2)	3.60 d (12.2)	3.61 d (12.3)	3.60 d (12.1)	3.60 d (12.2)	3.62 d (12.2)
2′	-	-	-	-	-	-
3′	5.53 d (8.1)	5.54 d (8.1)	5.49 d (7.8)	5.49 d (7.8)	5.49 d (7.8)	5.50 d (7.8)
4′	4.52 t (8.1)	4.52 t (8.1)	4.40 t (7.8)	4.40 t	4.39 t (7.8)	4.41 t (7.8)
5′	4.00 ddd (3.1/8.1/10.0)	4.00 ddd (3.1/8.1/10.0)	3.97 m	3.97 m	3.95 m	3.97 m
6′	3.91 dd (12.1/6.9) 3.84 †	3.90 dd (12.1/6.9) 3.84 †	3.85 †	3.85 †	3.86 †	3.86 †
Acyl→Glu-6(OH)	SA	SA	-	-	-	-
2″	6.88 s	6.84 s	-	-	-	-
6″	6.88 s	6.84 s	-	-	-	-
7″	7.57 d (15.7)	7.56 d (15.9)	-	-	-	-
8″	6.44 d (15.7)	6.44 d (15.9)	-	-	-	-
3″&5″-OMe	3.85 s	3.82 s	-	-		-
Acyl→Fru-3′(OH)	SA	TMC	MFA	SA	TMC	FA
2‴	6.84 s	6.90 s	7.22 d (2.0)	6.93 s	6.96 s	7.22 d (2.0)
5‴	-	-	6.95 d (8.0)	-	-	6.8 d (8.2)
6″″	6.84 s	6.90 s	7.21 dd (8.0/2.0)	6.93 s	6.96 s	7.14 dd (8.2/2.0)
7‴	7.65 d (15.7)	7.67 d (15.9)	7.71 d (15.9)	7.69 d (15.9)	7.72 d (16.0)	7.71 d (15.9)
8‴	6.42 d (15.7)	6.53 d (15.9)	6.47 d (15.9)	6.44 d (15.9)	6.54 d (16.0)	6.44 d (15.9)
3‴&5‴-OMe	3.82 s (6H)	3.84 s (6H)	3.85 s (3H)	3.87 s (6H)	3.88 s (6H)	3.89 s (3H)
4‴-OMe	-	3.77 s (3H)	3.86 s (3H)	-	3.80 s (3H)	-

SA = sinapic acid; TMC = trimethoxycinnamic acid; MFA = 3-*O*-methylferulic acid; FA = ferulic acid; † Signal patterns unclear due to overlapping.

**Table 2:** ^13^C-NMR data of sucrose esters (**1–6**) (δ_C_ 125 MHz, CD_3_OD).

		1	2	3	4	5	6
C/H	DEPT	δ_C_, ppm	δ_C_, ppm	δ_C_, ppm	δ_C_, ppm	δ_C_, ppm	δ_C_, ppm
Glu							
1	CH	92.59	92.61	93.19	93.18	93.23	93.20
2	CH	72.99	72.97	72.94	72.79	73.00	72.94
3	CH	75.00	75.01	74.86	74.87	74.93	74.42
4	CH	71.83	71.81	71.02	70.99	71.07	71.01
5	CH	72.41	72.42	74.42	74.42	74.51	74.87
6	CH_2_	65.60	65.58	62.20	62.20	62.28	62.18
Fru							
1′	CH_2_	63.72	63.72	65.19	65.20	65.24	65.19
2′	C	104.78	104.76	104.69	104.70	104.71	104.71
3′	CH	79.19	79.32	79.59	79.52	79.70	79.54
4′	CH	74.07	74.09	73.74	73.76	73.80	73.75
5′	CH	84.18	84.21	83.96	83.94	84.06	83.96
6′	CH_2_	65.60	65.58	62.84	62.82	62.85	62.84
Acyl→Glu-6(OH)		SA	SA				
1″	C	126.46	126.48				
2″	CH	106.89	106.75				
3″	C	149.25	149.24				
4″	C	139.42	139.33				
5″	C	149.25	149.24				
6″	CH	106.89	106.75				
7″	CH	147.33	147.23				
8″	CH	115.65	115.66				
9″	C	169.16	169.10				
3″&5″-OMe	CH_3_	56.79	56.73				
Acyl→Fru-3′(OH)		SA	TMC	MFA	SA	TMC	FA
1‴	C	126.51	131.34	128.56	126.49	131.46	127.60
2‴	CH	106.74	106.78	111.63	106.95	106.85	112.00
3″	C	149.24	154.59	150.47	149.27	154.66	150.49
4″″	C	139.30	141.10	152.68	139.43	141.17	149.21
5‴	C/CH	149.24	154.59	112.45	149.27	154.66	116.43
6‴	CH	106.74	106.78	124.10	106.95	106.85	124.15
7‴	CH	147.97	147.29	147.30	147.95	147.22	147.73
8‴	CH	115.29	117.63	115.83	115.32	117.74	114.89
9‴	C	168.31	167.85	168.17	168.26	167.82	168.39
3‴&5‴-OMe	CH_3_	56.84	56.79	56.51	56.90	56.79	56.88
4‴-OMe	CH_3_		61.18	61.39		61.20	

SA = sinapic acid; TMC = trimethoxycinnamic acid; MFA = 3-*O*-methylferulic acid; FA = ferulic acid.

**Table 3:** Results of cross-docking calculations for *Polygala*-derived phytochemicals, showing the JAMDA scores of the best docking solutions. MPO: myeloperoxidase; iNOS: inducible nitric oxide synthase; COX-2: cyclooxygenase-2.

Protein	Ligand	JAMDA Score	All-Atom RMSD
MPO (PDB ID: 5QJ2)	PDB chemical ID: JXS	−2.32663	0.632 Å
	Compound **1**	−3.78280	
	Compound **2**	−3.57348	
	Compound **3**	−2.90840	
	Compound **4**	−3.13930	
	Compound **5**	−2.97990	
	Compound **6**	−2.83140	
	Compound **7**	−2.50225	
	Compound **8**	−3.03640	
	Compound **9**	−3.52060	
iNOS (PDB ID: 3E7G)	PDB chemical ID: AT2	−2.62362	0.682 Å
	Compound **1**	−2.85444	
	Compound **2**	−3.14240	
	Compound **3**	−2.66980	
	Compound **4**	−2.34210	
	Compound **5**	−2.54230	
	Compound **6**	−3.02600	
	Compound **7**	−2.67380	
	Compound **8**	−3.47560	
	Compound **9**	−2.78550	
COX-2 (PDB ID: 5KIR)	PDB chemical ID: RCX	−2.52030	0.532 Å
	Compound **1**	Positive	
	Compound **2**	Positive	
	Compound **3**	Positive	
	Compound **4**	Positive	
	Compound **5**	Positive	
	Compound **6**	Positive	
	Compound **7**	−2.56960	
	Compound **8**	Positive	
	Compound **9**	Positive	

## Data Availability

The data that support the findings of this study are available upon reasonable request from the authors.

## Supplementary Materials

The following are available online. The HRMS and NMR spectra of compounds **1**–**9** are deposited as Supplementary Figures S1–S61.

## Sample Availability

The samples of compounds **1**–**9** are available upon reasonable request from the authors.
