# Supplementary material for: Isolation, Characterization and In Silico Studies of Secondary Metabolites from the Whole Plant of Polygala inexpectata Peşmen & Erik
Source: Molecules. 2022 Jan 21;27(3):684. doi: 10.3390/molecules27030684 (PMC8838668; doi:10.3390/molecules27030684)
Supplement: Supplementary file 1 [file molecules-27-00684-s001.zip › molecules-1548138-supplementary.pdf]

Supplementary Material

Isolation, Characterization and *In Silico* Studies of Secondary Metabolites from the Whole Plant of *Polygala inexpectata* Peşmen & Erik

Ayşe Ünlü[1*], Kerem Teralı[2], Zübeyde Uğurlu Aydın[1], Ali A. Dönmez[1], Hasan Soliman Yusufoğlu[3], İhsan Çalış[4]

[1] Department of Biology, Faculty of Science, Hacettepe University, Beytepe, Ankara, Turkey; ayshe_hsn@hotmail.com
[2] Department of Medical Biochemistry, Faculty of Medicine, Girne American University, Girne, TRNC; keremterali@gau.edu.tr
[3] Department of Pharmacognosy, Prince Sattam Bin Abdulaziz University, Al-Kharj 11942, Saudi Arabia; yusufoglu@psau.edu.sa
[4] Department of Pharmacognosy, Facuty of Pharmacy, Near East University, Lefkoşa, TRNC; ihsan.calis@neu.edu.tr

* Corresponding author: Ayşe Ünlü

E-mail address: ayshe_hsn@hotmail.com

List of Supplementary Data

Figure S1. 6,3'-disinapoyl-sucrose (1) ... 4
Figure S2. (+) HRMS of **6,3'-disinapoyl-sucrose (1)** ... 4
Figure S3. ^{1}H-NMR spectrum of **6,3'-disinapoyl-sucrose (1)** (500 MHz, CD_3OD) ... 5
Figure S4. ^{13}C-NMR spectrum of **6,3'-disinapoyl-sucrose (1)** (125 MHz, CD_3OD) ... 5
Figure S5. DEP135 of **6,3'-disinapoyl-sucrose (1)** ... 5
Figure S6. COSY of **6,3'-disinapoyl-sucrose (1)** ... 5
Figure S7. HSQC of **6,3'-disinapoyl-sucrose (1)** ... 6
Figure S8. HMBC of **6,3'-disinapoyl-sucrose (1)** ... 6
Figure S9. 6-*O*-sinapoyl,3'-*O*-trimethoxy-cinnamoyl-sucrose (tenuifoliside C) (2) ... 7
Figure S10. (+)-HRMS of **6-*O*-sinapoyl,3'-*O*-trimethoxy-cinnamoyl-sucrose (tenuifoliside C) (2)** ... 7
Figure S11. ^{1}H-NMR Spectrum of **6-*O*-sinapoyl,3'-*O*-trimethoxy-cinnamoyl-sucrose (tenuifoliside C) (2)** (500 MHz, CD_3OD) ... 7
Figure S12. ^{13}C-NMR Spectrum of **6-*O*-sinapoyl,3'-*O*-trimethoxy-cinnamoyl-sucrose (tenuifoliside C) (2)** (125 MHz, CD_3OD) ... 8
Figure S13. DEPT-135 of **6-*O*-sinapoyl,3'-*O*-trimethoxy-cinnamoyl-sucrose (tenuifoliside C) (2)** ... 8
Figure S14. COSY of **6-*O*-sinapoyl,3'-*O*-trimethoxy-cinnamoyl-sucrose (tenuifoliside C) (2)** ... 9
Figure S15. HSQC of **6-*O*-sinapoyl,3'-*O*-trimethoxy-cinnamoyl-sucrose (tenuifoliside C) (2)** ... 9
Figure S16. HMBC of **6-*O*-sinapoyl,3'-*O*-trimethoxy-cinnamoyl-sucrose (tenuifoliside C) (2)** ... 10
Figure S17. 3'-*O*-(*O*-methyl-feruloyl)-sucrose (3) ... 10
Figure S18. (+)-HRMS of **3'-*O*-(*O*-methyl-feruloyl)-sucrose (3)** ... 10
Figure S19. ^{1}H-NMR Spectrum of **3'-*O*-(*O*-methyl-feruloyl)-sucrose (3)** (500 MHz, CD_3OD) ... 11
Figure S20. ^{13}C-NMR Spectrum of **3'-*O*-(*O*-methyl-feruloyl)-sucrose (3)** (125 MHz, CD_3OD) ... 11
Figure S21. DEPT-135 of **3'-*O*-(*O*-methyl-feruloyl)-sucrose (3)** ... 12
Figure S22. COSY of **3'-*O*-(*O*-methyl-feruloyl)-sucrose (3)** ... 12
Figure S23. HSQC of **3'-*O*-(*O*-methyl-feruloyl)-sucrose (3)** ... 13
Figure S24. HMBC of **3'-*O*-(*O*-methyl-feruloyl)-sucrose (3)** ... 13
Figure S25. 3'-*O*-(sinapoyl)-sucrose (4) ... 14
Figure S26. (+)-HRMS of **3'-*O*-(sinapoyl)-sucrose (4)** ... 14
Figure S27. ^{1}H-NMR Spectrum of **3'-*O*-(sinapoyl)-sucrose (4)** (500 MHz, CD_3OD) ... 14
Figure S28. ^{13}C-NMR Spectrum of **3'-*O*-(sinapoyl)-sucrose (4)** (125 MHz, CD_3OD) ... 15
Figure S29. DEPT-135 of **3'-*O*-(sinapoyl)-sucrose (4)** ... 15
Figure S30. HMBC of **3'-*O*-(sinapoyl)-sucrose (4)** ... 15
Figure S31. 3'-*O*-trimethoxy-cinnamoyl-sucrose (glomeratose) (5) ... 16
Figure S32. (+)-HRMS of **3'-*O*-trimethoxy-cinnamoyl-sucrose (glomeratose) (5)** ... 16
Figure S33. ^{1}H-NMR Spectrum of **3'-*O*-trimethoxy-cinnamoyl-sucrose (glomeratose) (5)** (500 MHz, CD_3OD) ... 16
Figure S34. ^{13}C-NMR Spectrum of **3'-*O*-trimethoxy-cinnamoyl-sucrose (glomeratose) (5)** (125 MHz, CD_3OD) ... 17
Figure S35. DEPT-135 of **3'-*O*-trimethoxy-cinnamoyl-sucrose (glomeratose) (5)** ... 17
Figure S36. COSY of **3'-*O*-trimethoxy-cinnamoyl-sucrose (glomeratose) (5)** ... 17
Figure S37. HSQC of **3'-*O*-trimethoxy-cinnamoyl-sucrose (glomeratose) (5)** ... 18
Figure S38. HMBC of **3'-*O*-trimethoxy-cinnamoyl-sucrose (glomeratose) (5)** ... 18
Figure S39. 3'-*O*-feruloyl-sucrose (sibiricose A5) (6) ... 19

Figure S40. (+)-HRMS of **3'-*O*-feruloyl-sucrose (sibiricose A5) (6)** 19
Figure S41. ^{1}H-NMR spectrum of **3'-*O*-feruloyl-sucrose (sibiricose A5) (6)** (500 MHz, CD_3OD) 19
Figure S42. ^{13}C-NMR spectrum of **3'-*O*-feruloyl-sucrose (sibiricose A5) (6)** (125 MHz, CD_3OD) 20
Figure S43. DEPT-135 of **3'-*O*-feruloyl-sucrose (sibiricose A5) (6)** 20
Figure S44. COSY of **3'-*O*-feruloyl-sucrose (sibiricose A5) (6)** 20
Figure S45. HSQC of **3'-*O*-feruloyl-sucrose (sibiricose A5) (6)** 21
Figure S46. HMBC of **3'-*O*-feruloyl-sucrose (sibiricose A5) (6)** 21
Figure S47 sinapyl alcohol 4-*O*-glucoside (syringin or eleutheroside B) (7) 22
Figure S48. (+)-HRMS of **sinapyl alcohol 4-*O*-glucoside (syringin or eleutheroside B) (7)** 22
Figure S49. ^{1}H-NMR Spectrum of **sinapyl alcohol 4-*O*-glucoside (syringin or eleutheroside B) (7)** (500 MHz, CD_3OD) 22
Figure S50. ^{13}C-NMR Spectrum of **sinapyl alcohol 4-*O*-glucoside (syringin or eleutheroside B) (7)** (125 MHz, CD_3OD) 23
Figure S51. DEPT-135 of **sinapyl alcohol 4-*O*-glucoside (syringin or eleutheroside B) (7)** 23
Figure S52. liriodendrin (8) 23
Figure S53. (+)-HRMS of **liriodendrin (8)** 24
Figure S54. ^{1}H-NMR spectrum of **liriodendrin (8)** (500 MHz, CD_3OD) 24
Figure S55. ^{13}C-NMR spectrum of **liriodendrin (8)** (125 MHz, CD_3OD) 24
Figure S56. DEPT-135 of **liriodendrin (8)** 24
Figure S57. 7,4'-di-*O*-methylquercetin-3-*O*-β-rutinoside (ombuin 3-*O*-rutinoside or ombuoside) (9) .. 25
Figure S58. (+)-HRMS of **7,4'-di-*O*-methylquercetin-3-*O*-β-rutinoside (ombuin 3-*O*-rutinoside or ombuoside) (9)** 25
Figure S59. ^{1}H-NMR Spectrum of **7,4'-di-*O*-methylquercetin-3-*O*-β-rutinoside (ombuin 3-*O*-rutinoside or ombuoside) (9)** (500 MHz, CD_3OD) 25
Figure S60. ^{13}C-NMR Spectrum of **7,4'-di-*O*-methylquercetin-3-*O*-β-rutinoside (ombuin 3-*O*-rutinoside or ombuoside) (9)** 26
Figure S61. DEPT-135 of **7,4'-di-*O*-methylquercetin-3-*O*-β-rutinoside (ombuin 3-*O*-rutinoside or ombuoside) (9)** 26

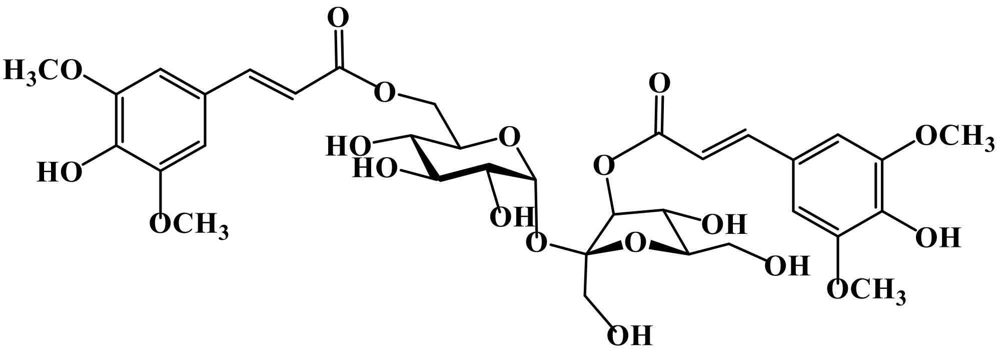

Exact Mass: 754,2320

Figure S1. 6,3'-disinapoyl-sucrose (1)

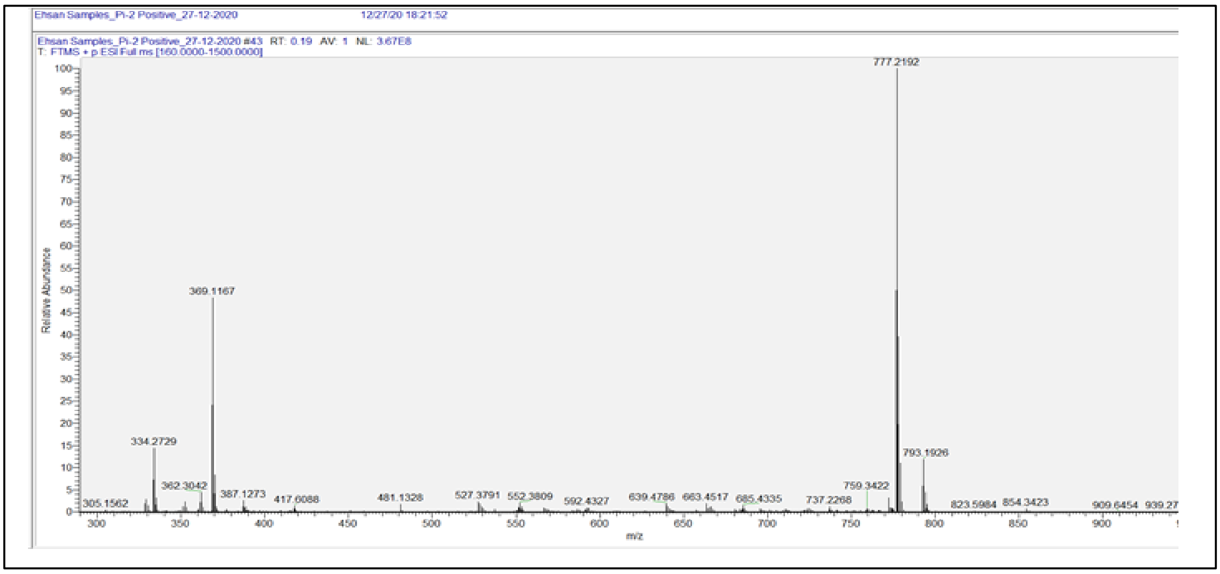

Figure S2. (+) HRMS of **6,3'-disinapoyl-sucrose (1)**

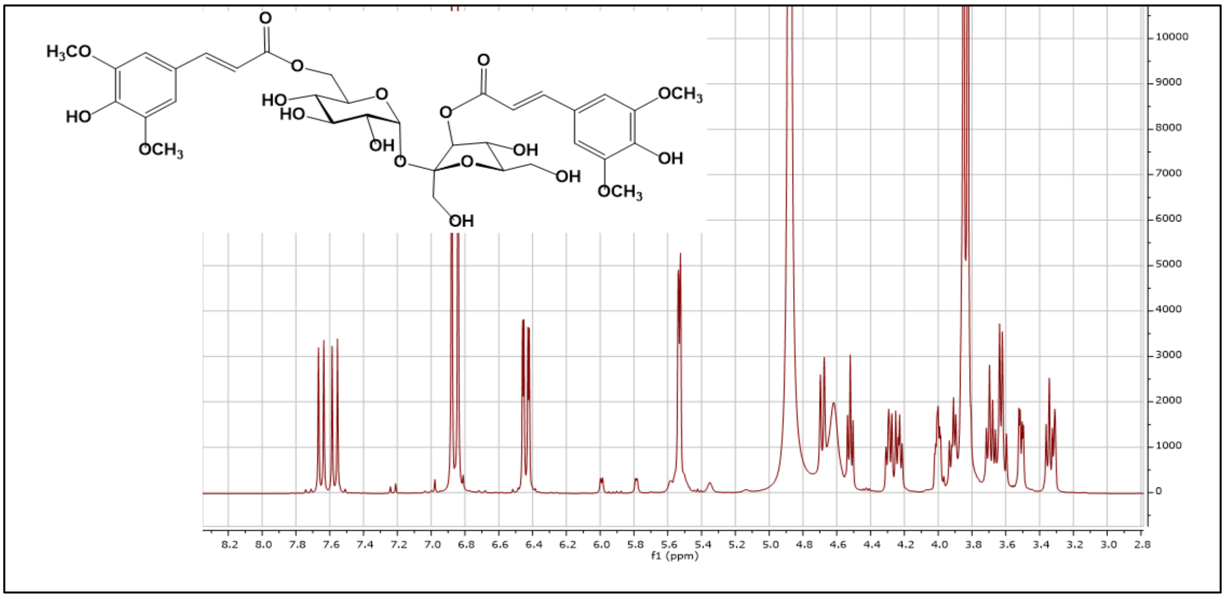

Figure S3. ^{1}H-NMR spectrum of **6,3'-disinapoyl-sucrose (1)** (500 MHz, CD_3OD)

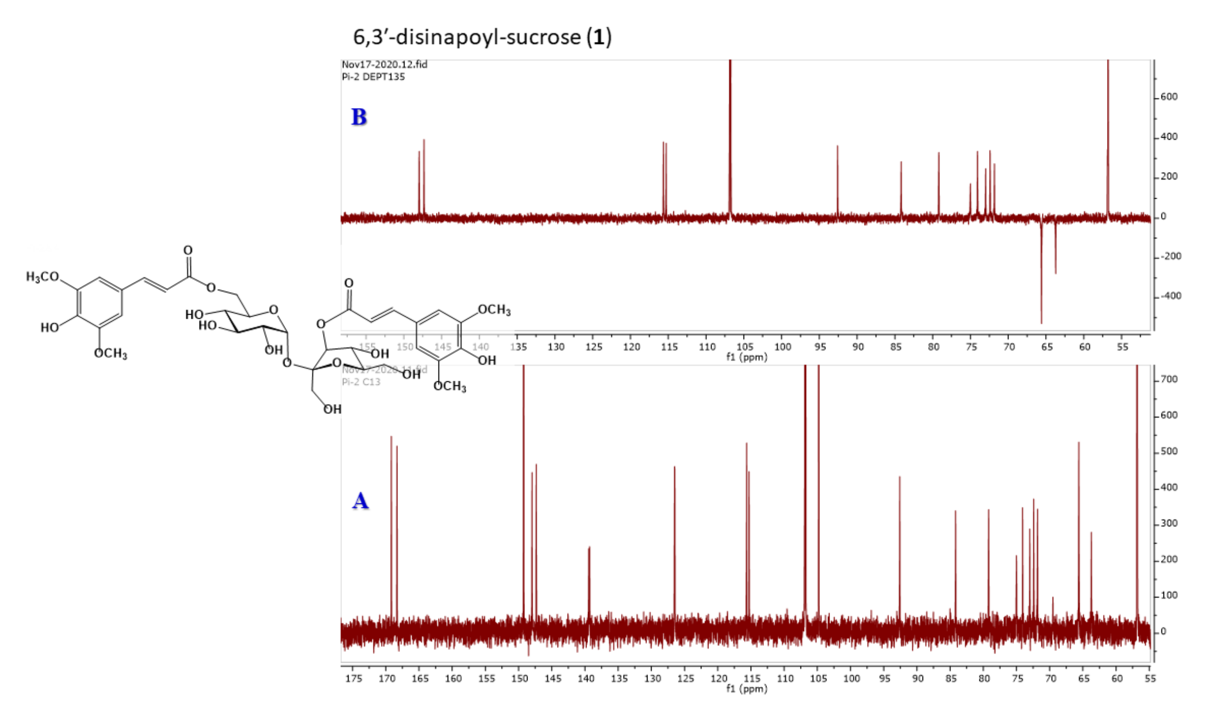

Figures S4&S5. ^{13}C-NMR spectrum (A) and DEPT-135 (B) of **6,3'-disinapoyl-sucrose (1)** (125 MHz, CD_3OD)

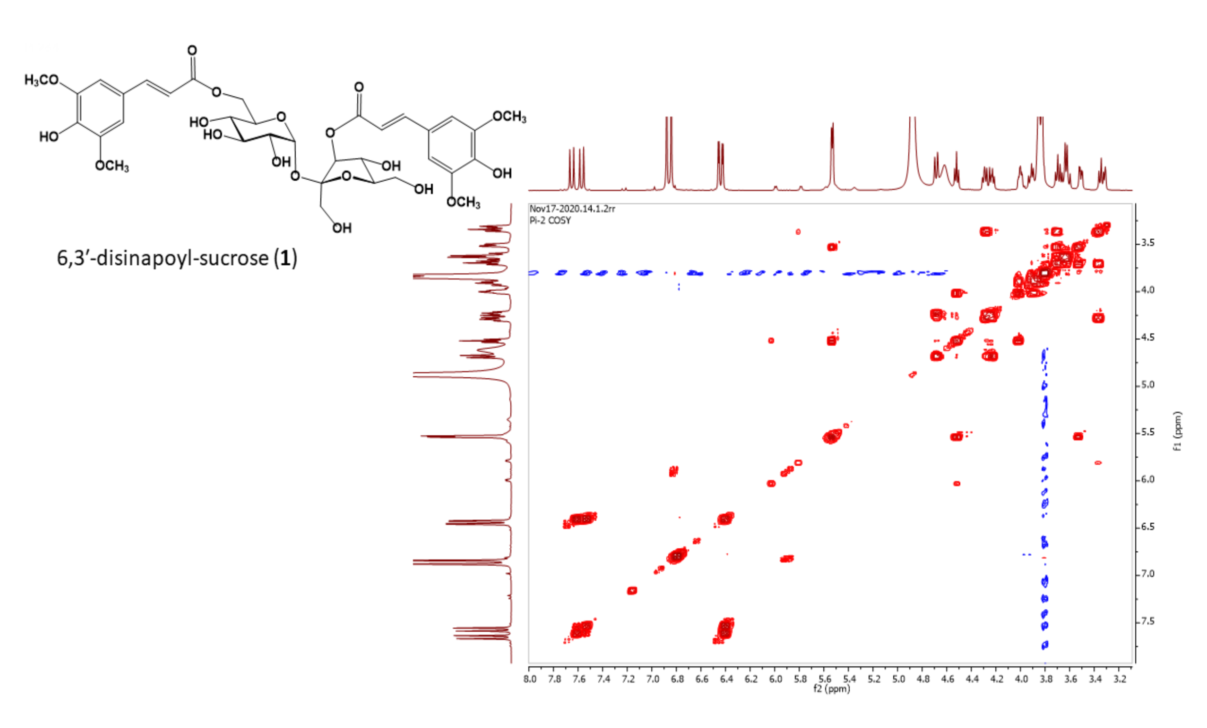

Figure S6. COSY of **6,3'-disinapoyl-sucrose (1)**

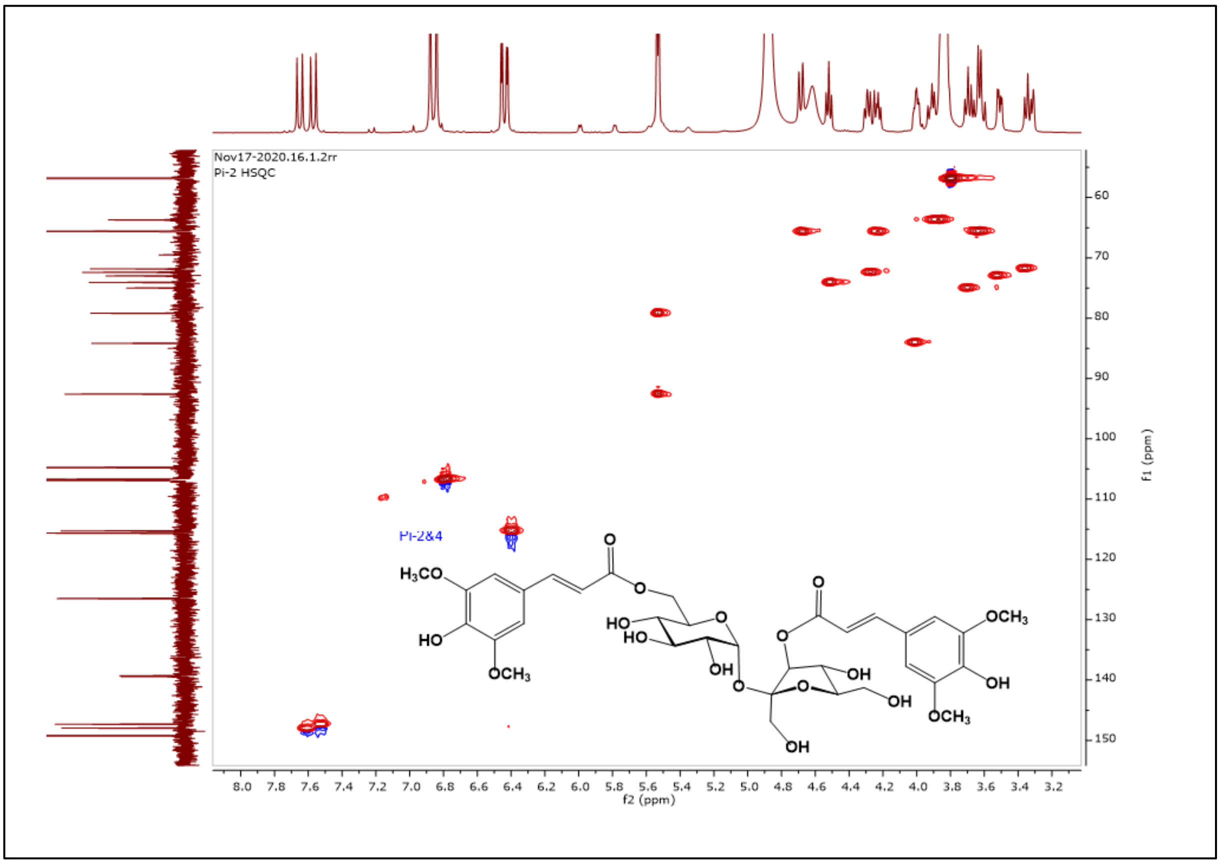

Figure S7. HSQC of **6,3'-disinapoyl-sucrose (1)**

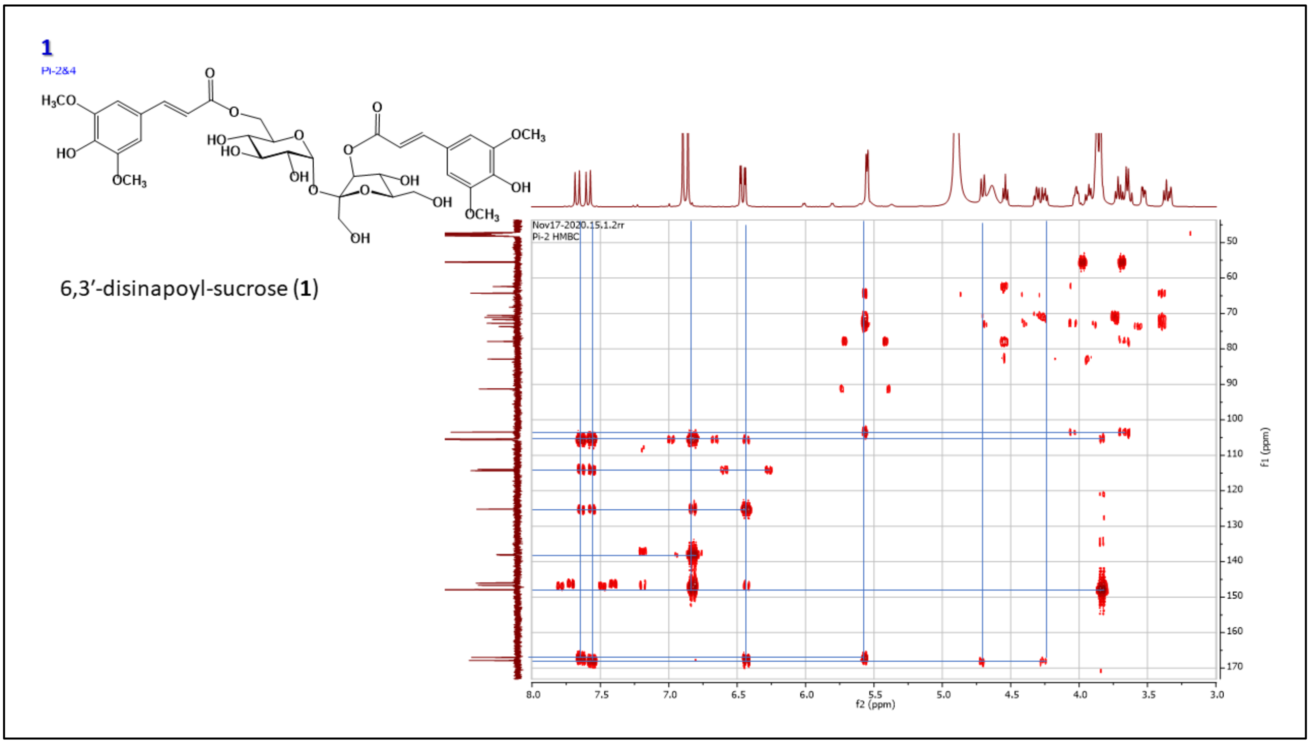

Figure S8. HMBC of **6,3'-disinapoyl-sucrose (1)**

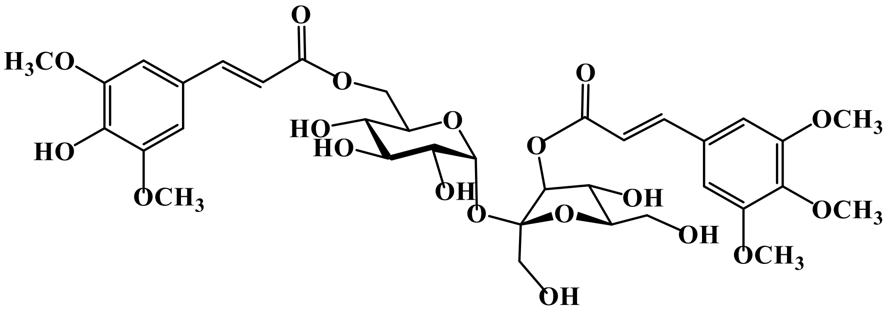

Exact Mass: 768,2477

Figure S9. 6-*O*-sinapoyl,3'-*O*-trimethoxy-cinnamoyl-sucrose (tenuifoliside C) (2)

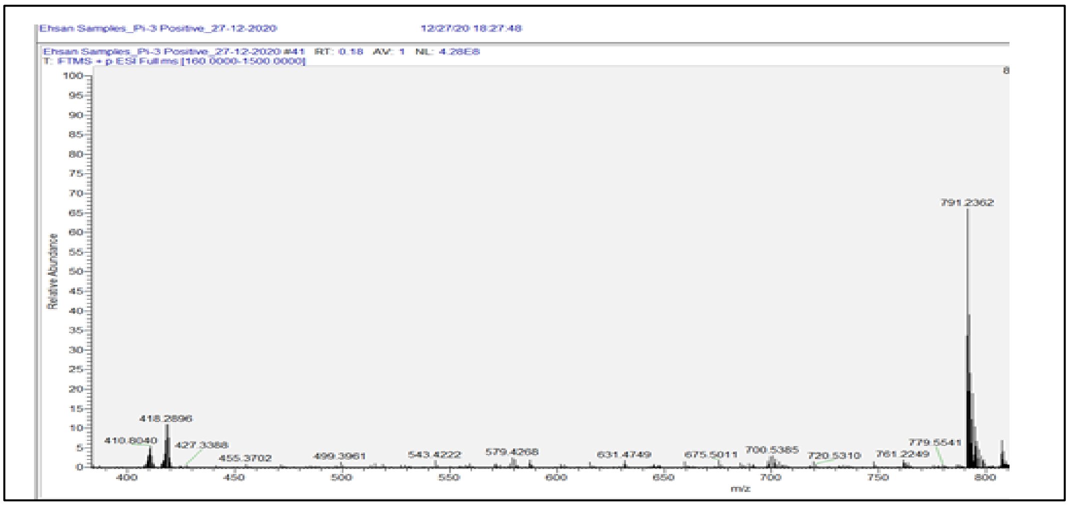

Figure S10. (+)-HRMS of **6-*O*-sinapoyl,3'-*O*-trimethoxy-cinnamoyl-sucrose (tenuifoliside C) (2)**

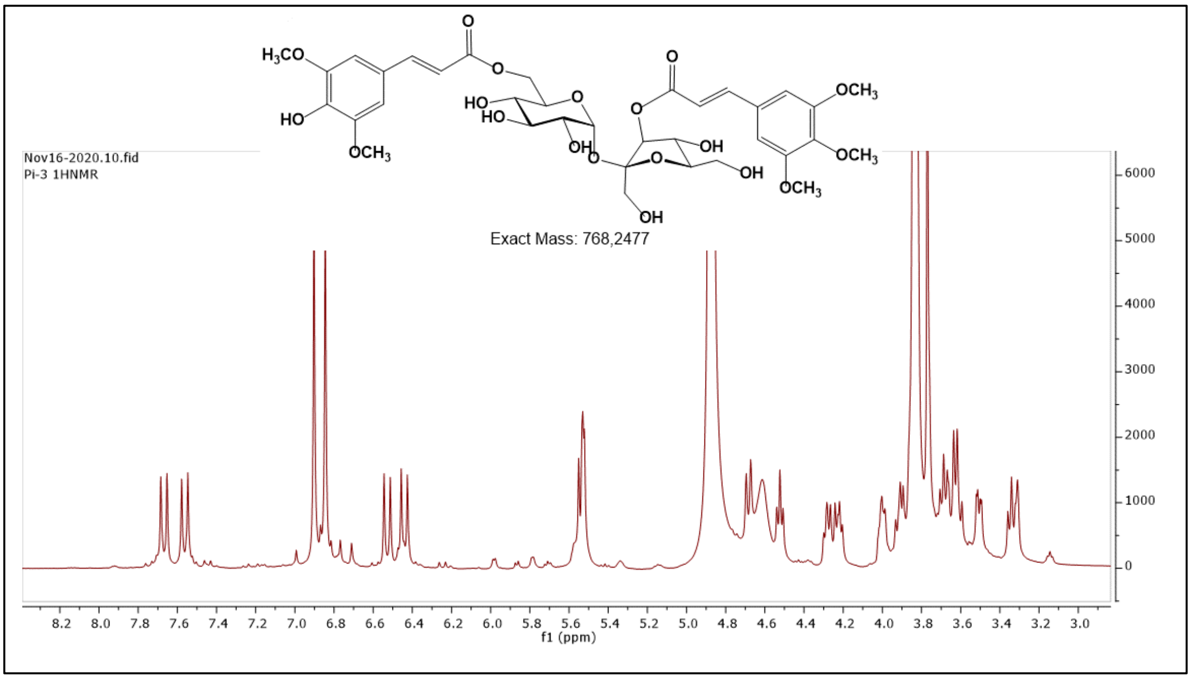

Figure S11. ^{1}H-NMR Spectrum of **6-*O*-sinapoyl,3'-*O*-trimethoxy-cinnamoyl-sucrose (tenuifoliside C) (2)** (500 MHz, CD_3OD)

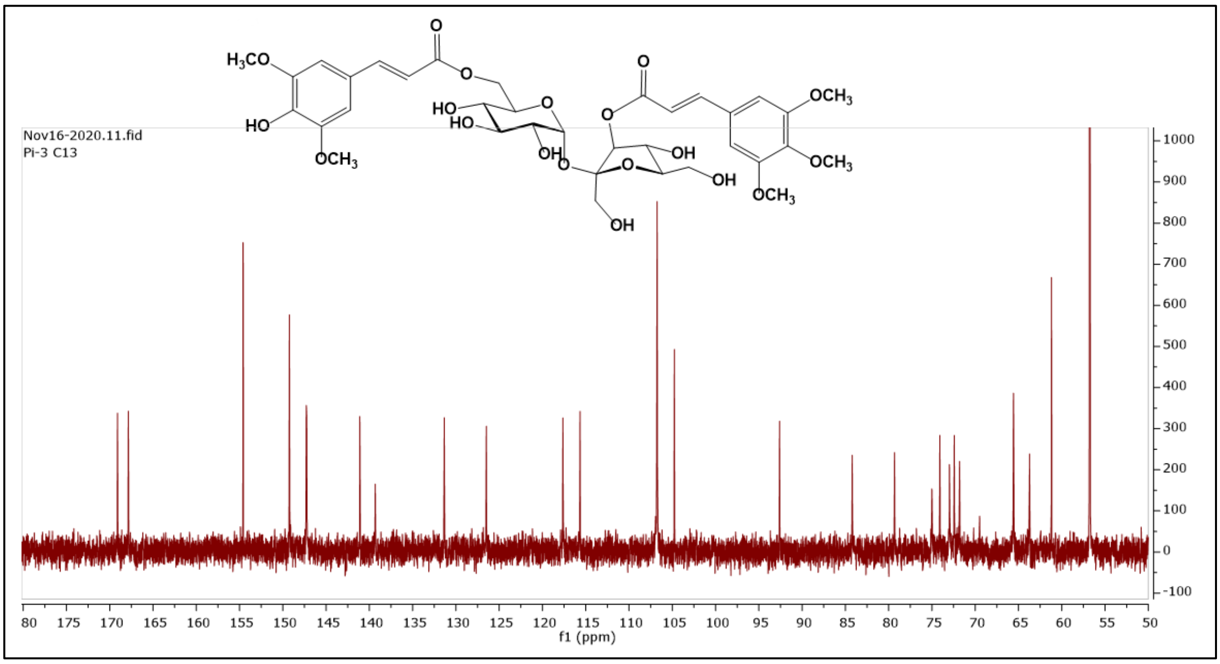

Figure S12. ^{13}C-NMR Spectrum of **6-*O*-sinapoyl,3'-*O*-trimethoxy-cinnamoyl-sucrose (tenuifoliside C) (2)** (125 MHz, CD_3OD)

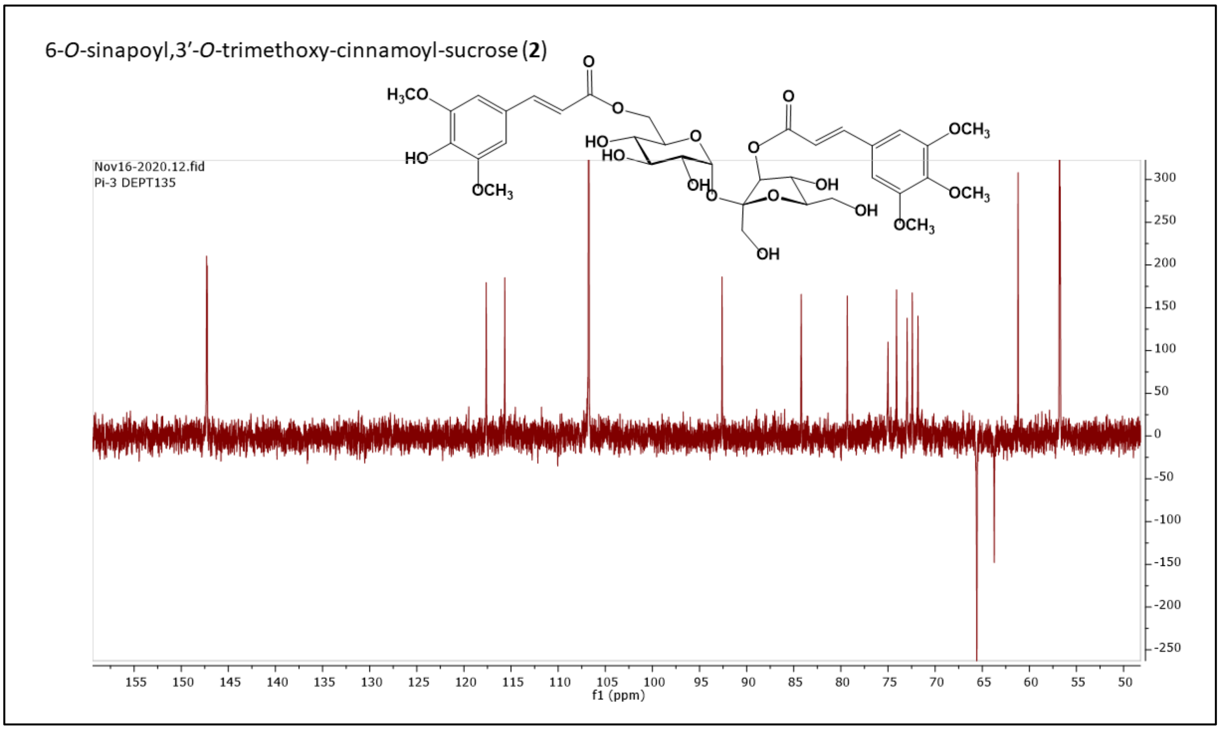

Figure S13. DEPT-135 of **6-*O*-sinapoyl,3'-*O*-trimethoxy-cinnamoyl-sucrose (tenuifoliside C) (2)**

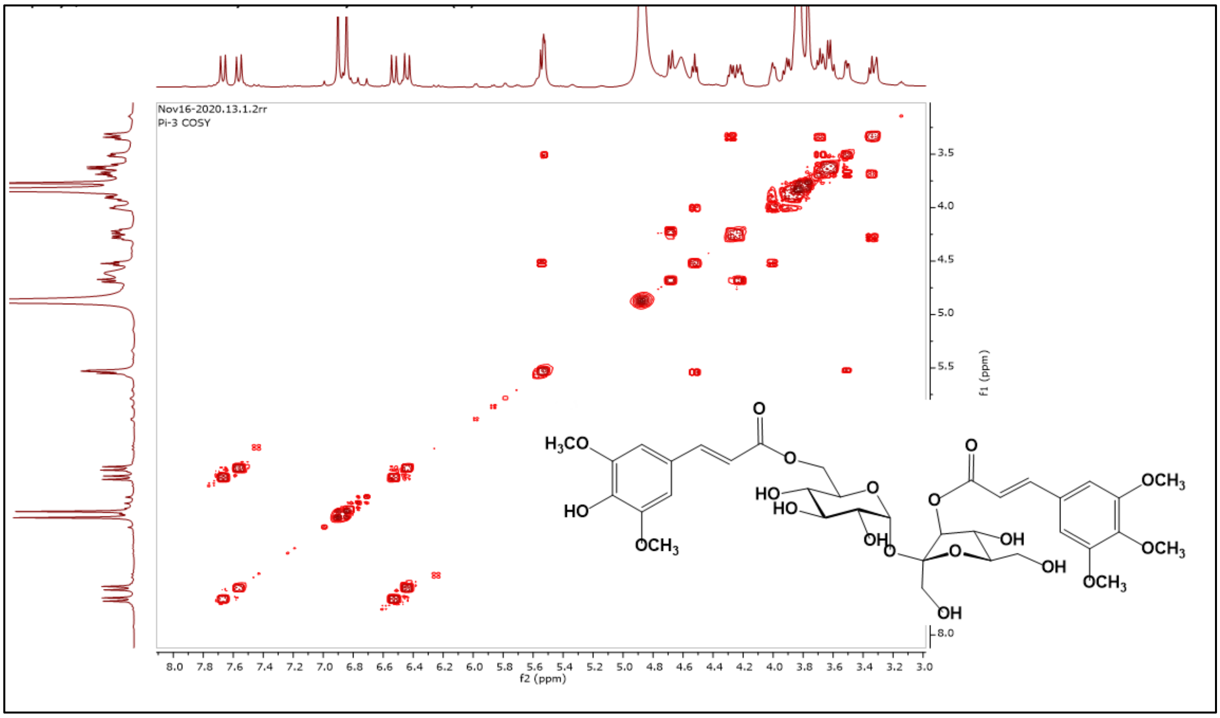

Figure S14. COSY of **6-*O*-sinapoyl,3'-*O*-trimethoxy-cinnamoyl-sucrose (tenuifoliside C) (2)**

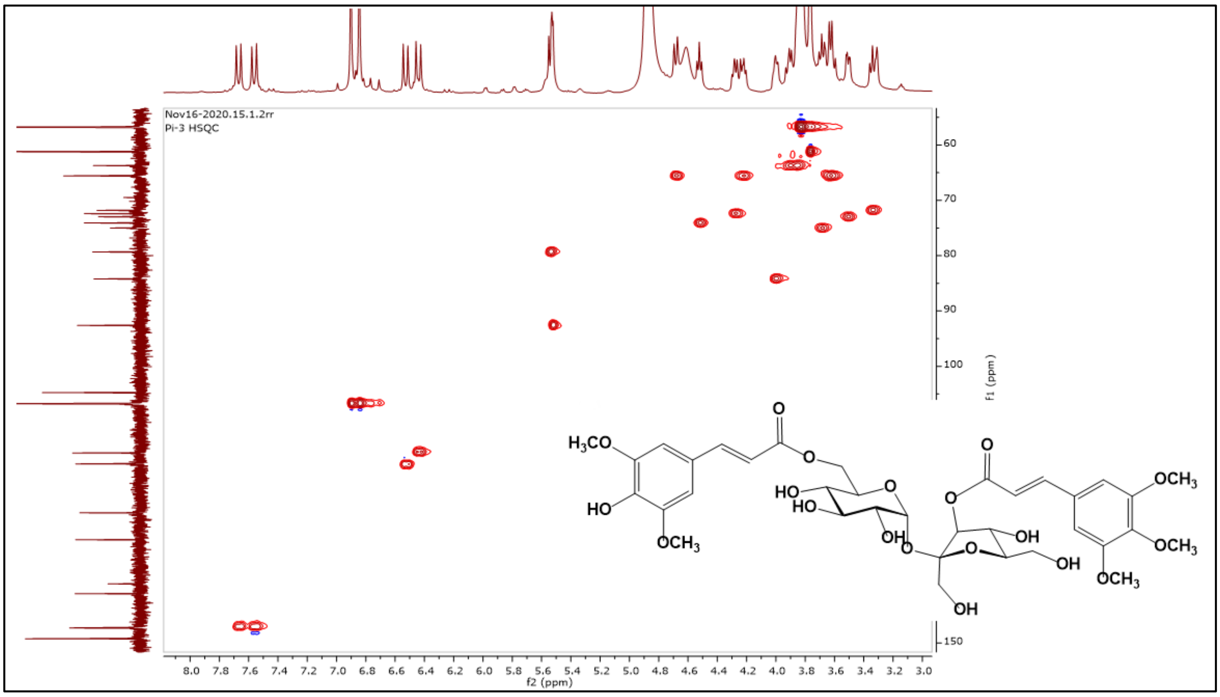

Figure S15. HSQC of **6-*O*-sinapoyl,3'-*O*-trimethoxy-cinnamoyl-sucrose (tenuifoliside C) (2)**

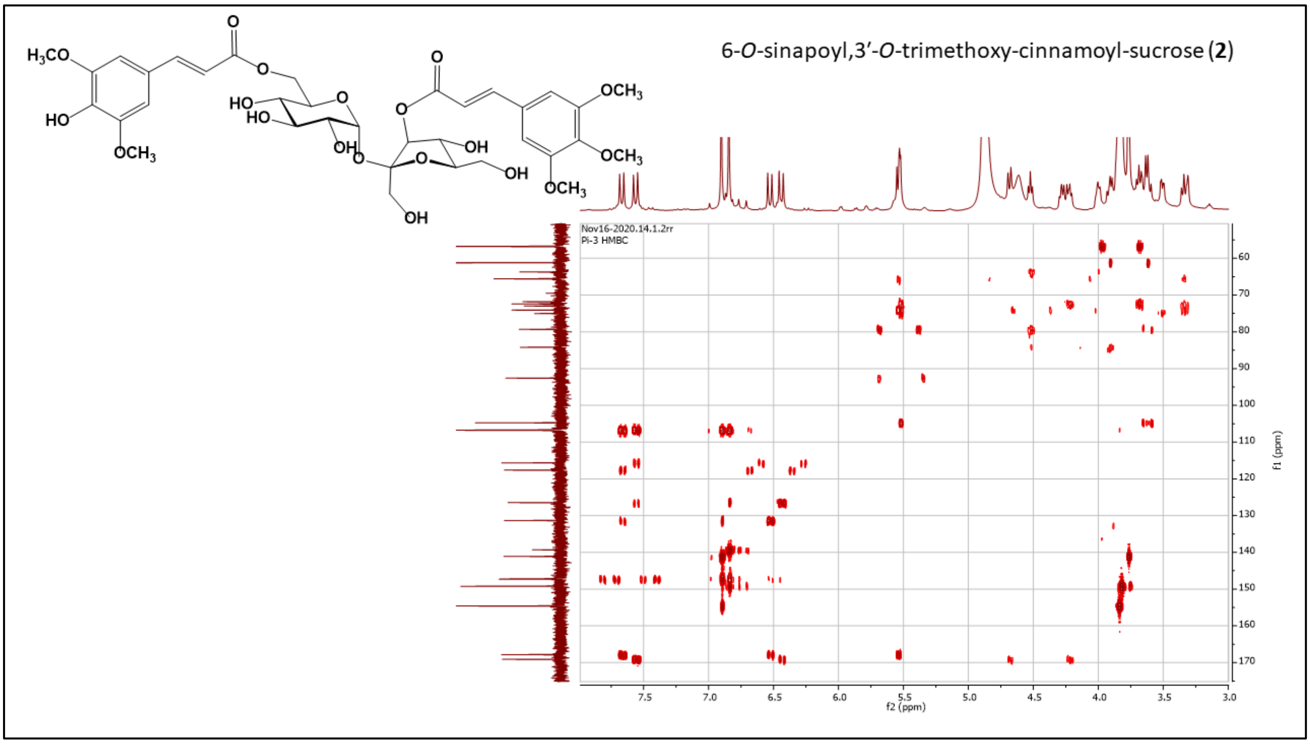

Figure S16. HMBC of **6-*O*-sinapoyl,3'-*O*-trimethoxy-cinnamoyl-sucrose (tenuifoliside C) (2)**

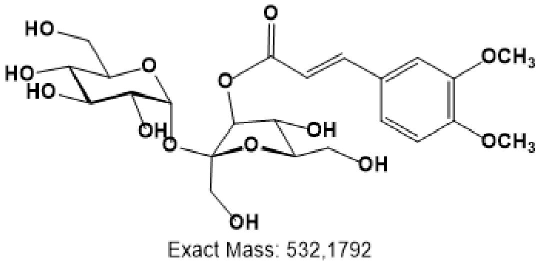

Figure S17. 3'-*O*-(*O*-methyl-feruloyl)-sucrose (3)

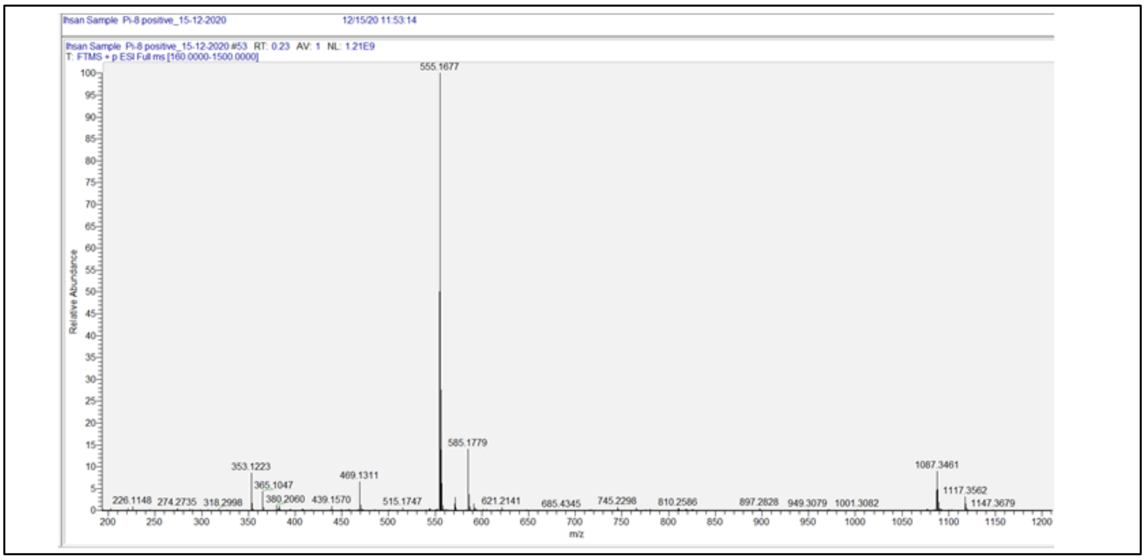

Figure S18. (+)-HRMS of **3'-*O*-(*O*-methyl-feruloyl)-sucrose (3)**

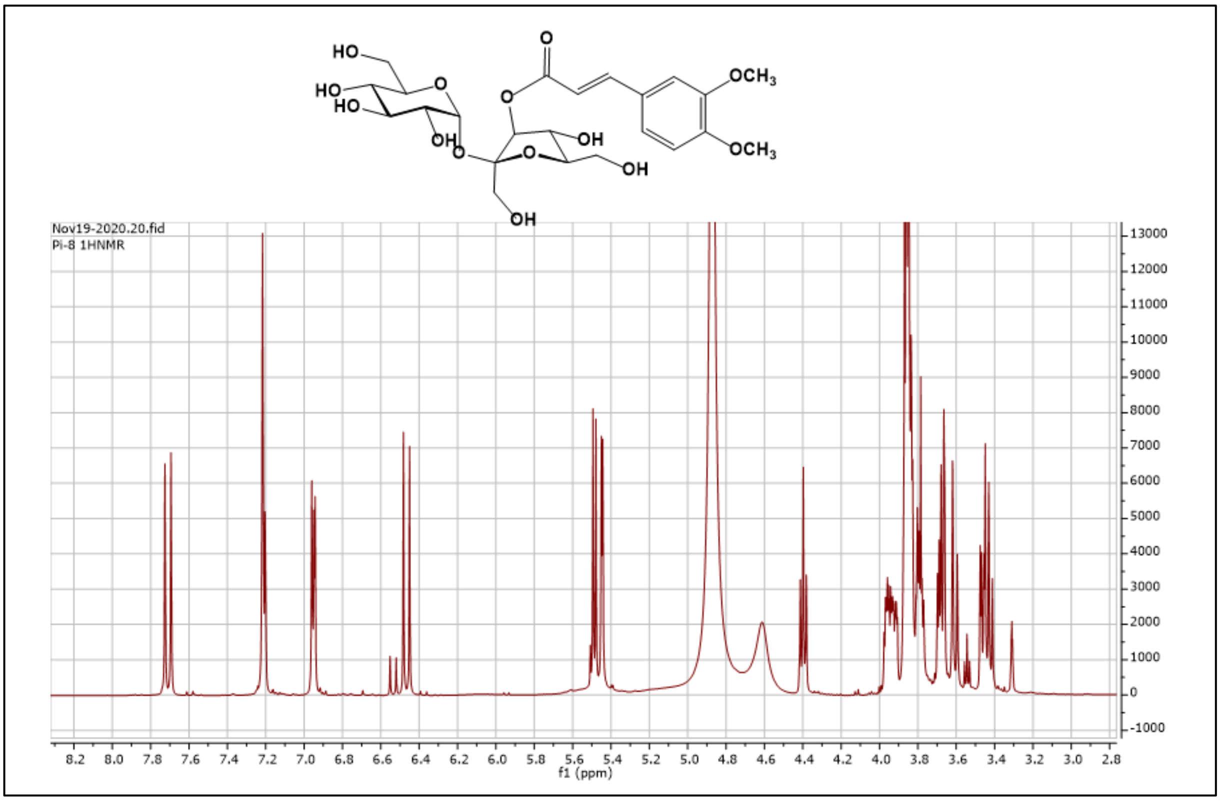

Figure S19. ^{1}H-NMR Spectrum of **3'-*O*-(*O*-methyl-feruloyl)-sucrose (3)** (500 MHz, CD_3OD)

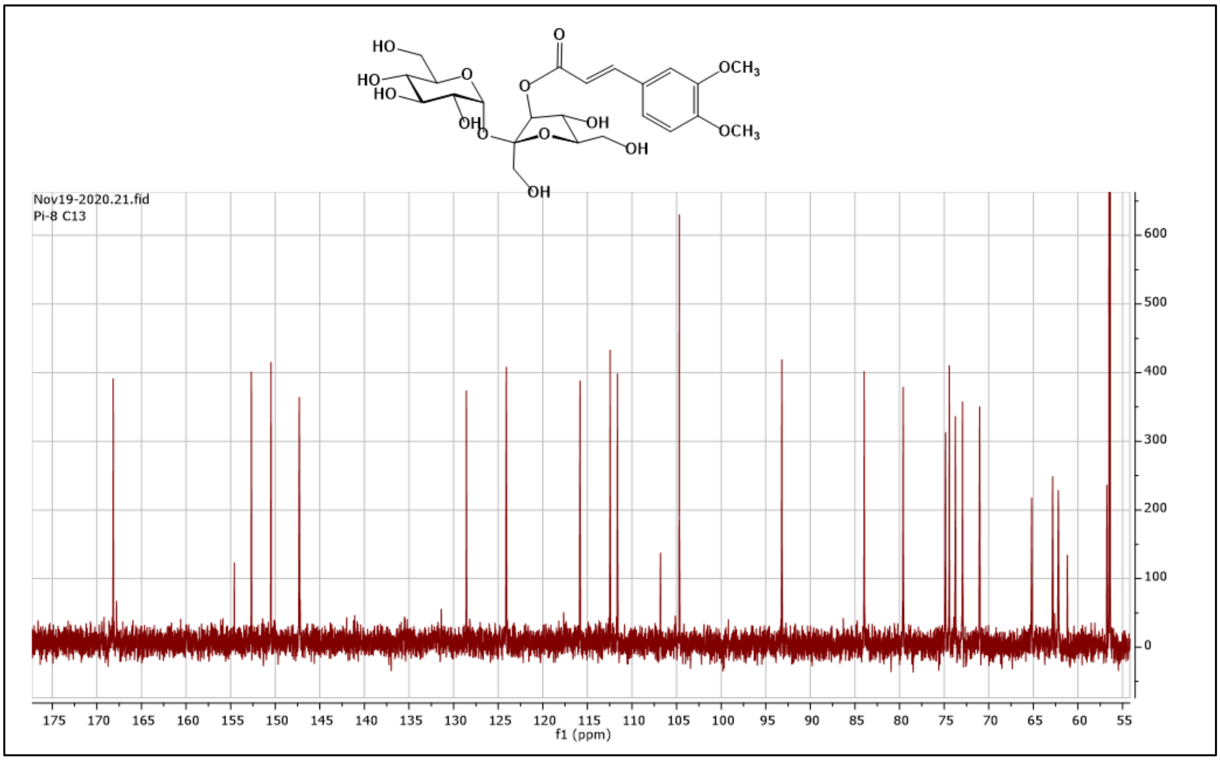

Figure S20. ^{13}C-NMR Spectrum of **3'-*O*-(*O*-methyl-feruloyl)-sucrose (3)** (125 MHz, CD_3OD)

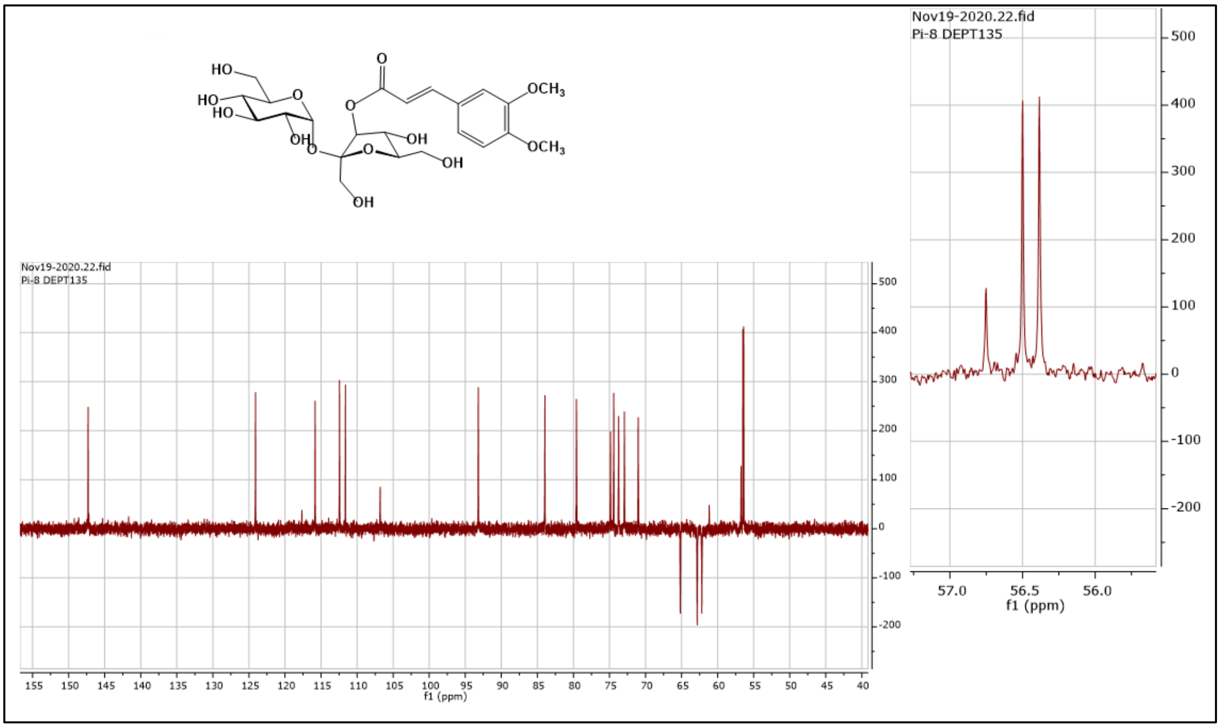

Figure S21. DEPT-135 of **3'-*O*-(*O*-methyl-feruloyl)-sucrose (3)**

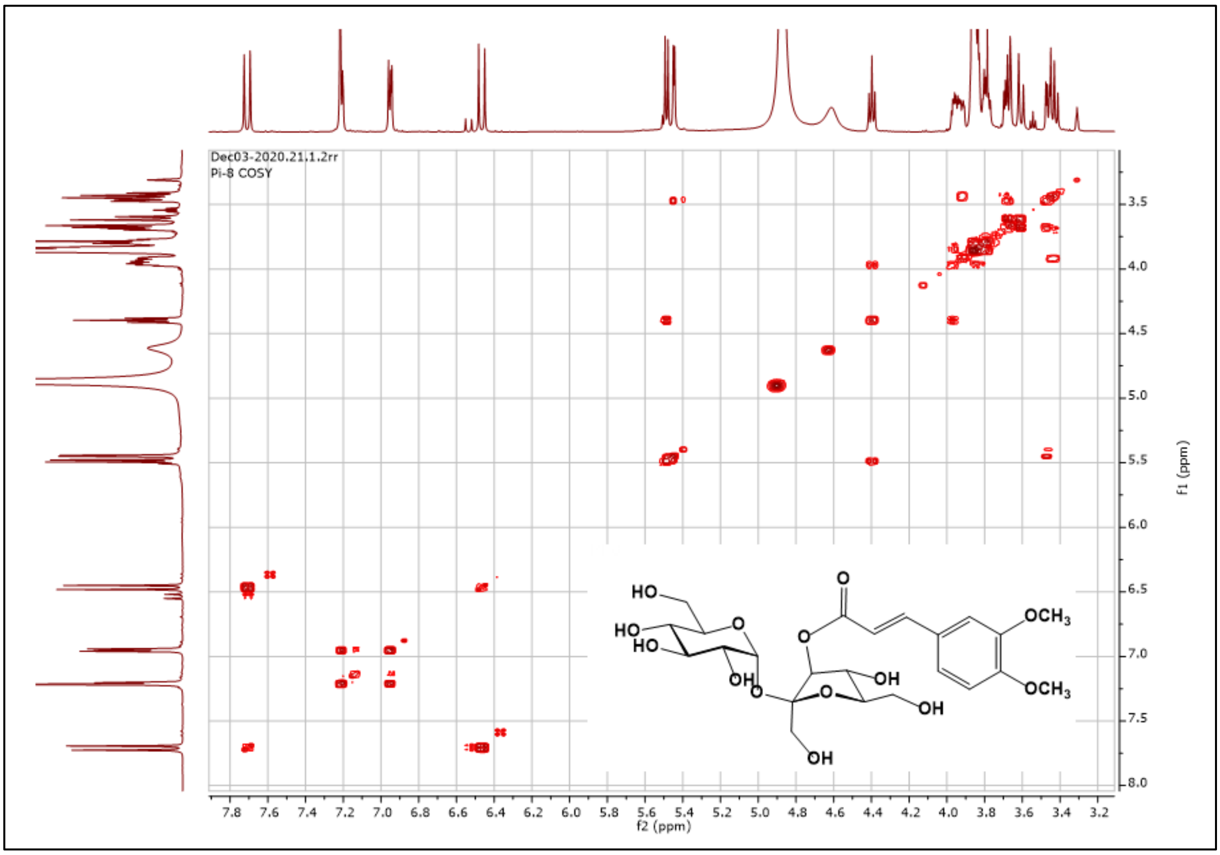

Figure S22. COSY of **3'-*O*-(*O*-methyl-feruloyl)-sucrose (3)**

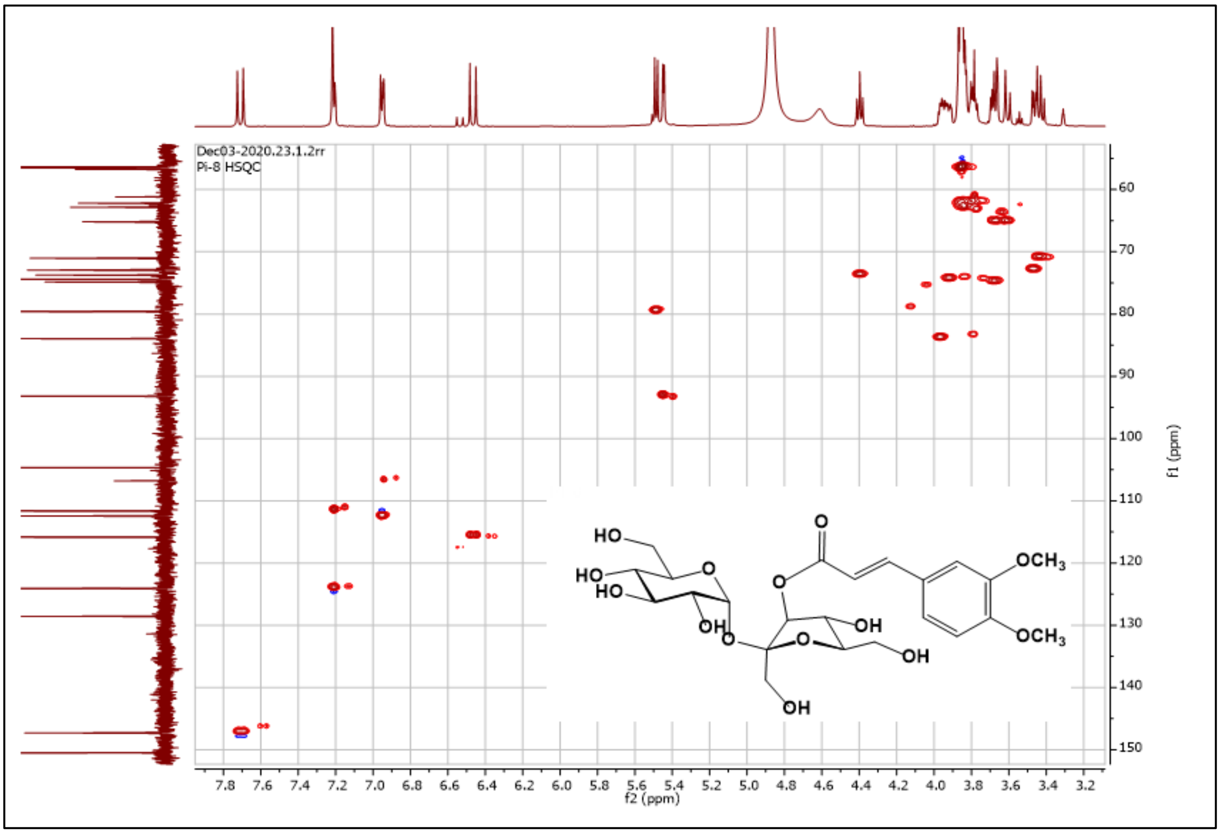

Figure S23. HSQC of **3'-*O*-(*O*-methyl-feruloyl)-sucrose (3)**

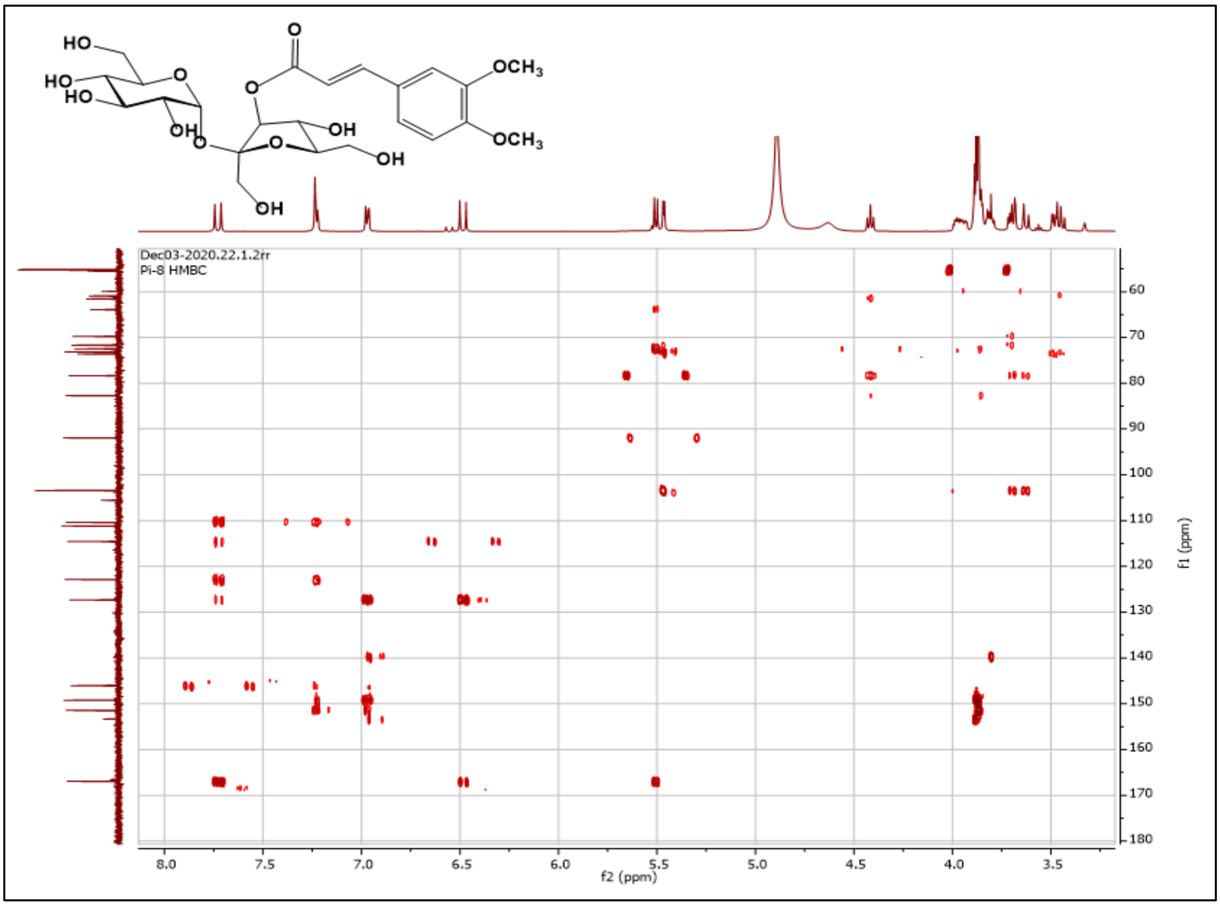

Figure S24. HMBC of **3'-*O*-(*O*-methyl-feruloyl)-sucrose (3)**

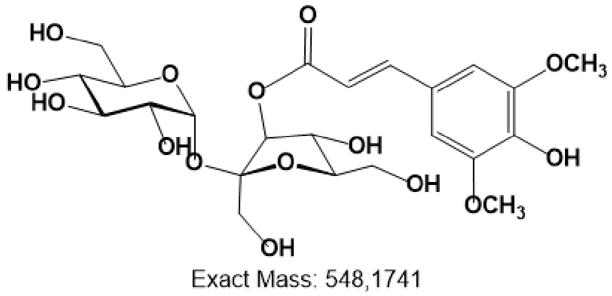

Figure S25. 3'-*O*-(sinapoyl)-sucrose (4)

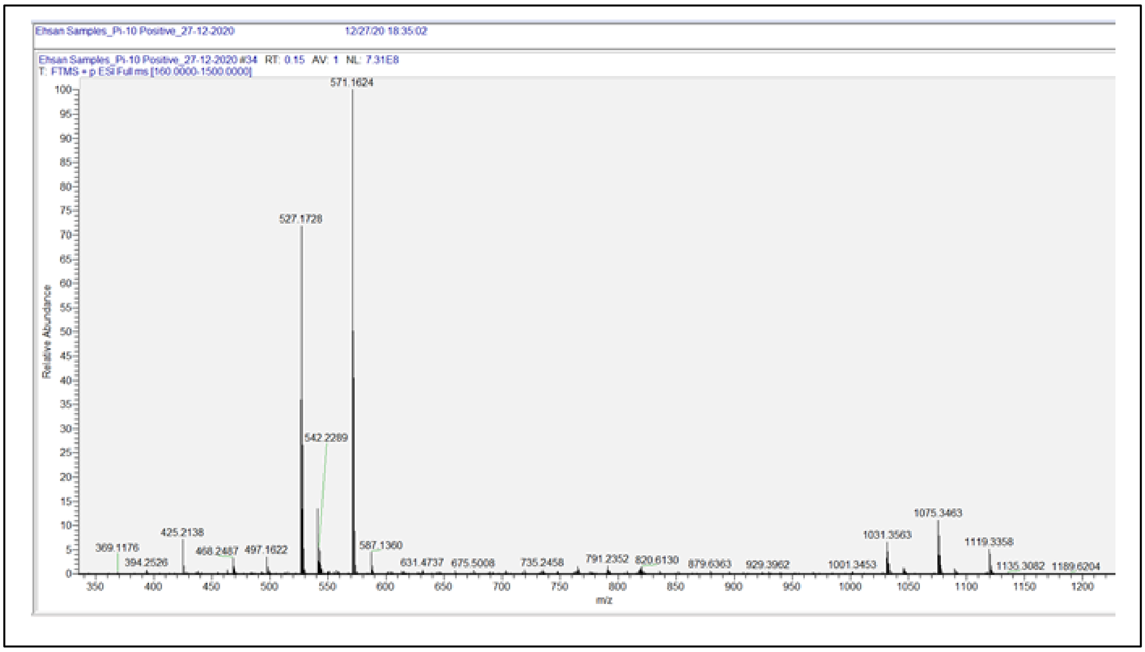

Figure S26. (+)-HRMS of **3'-*O*-(sinapoyl)-sucrose (4)**

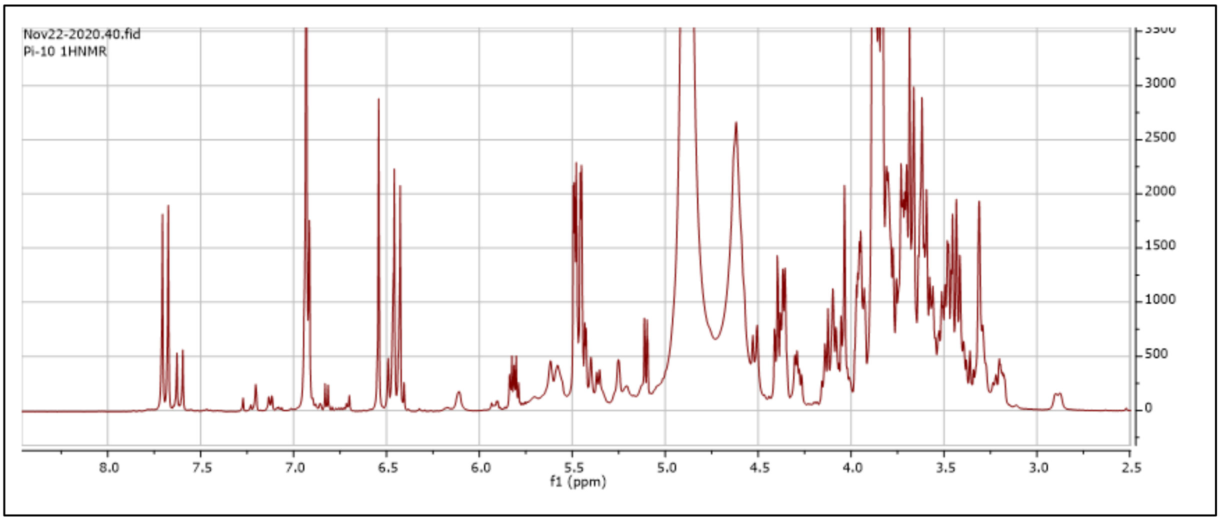

Figure S27. ^{1}H-NMR Spectrum of **3'-*O*-(sinapoyl)-sucrose (4)** (500 MHz, CD_3OD)

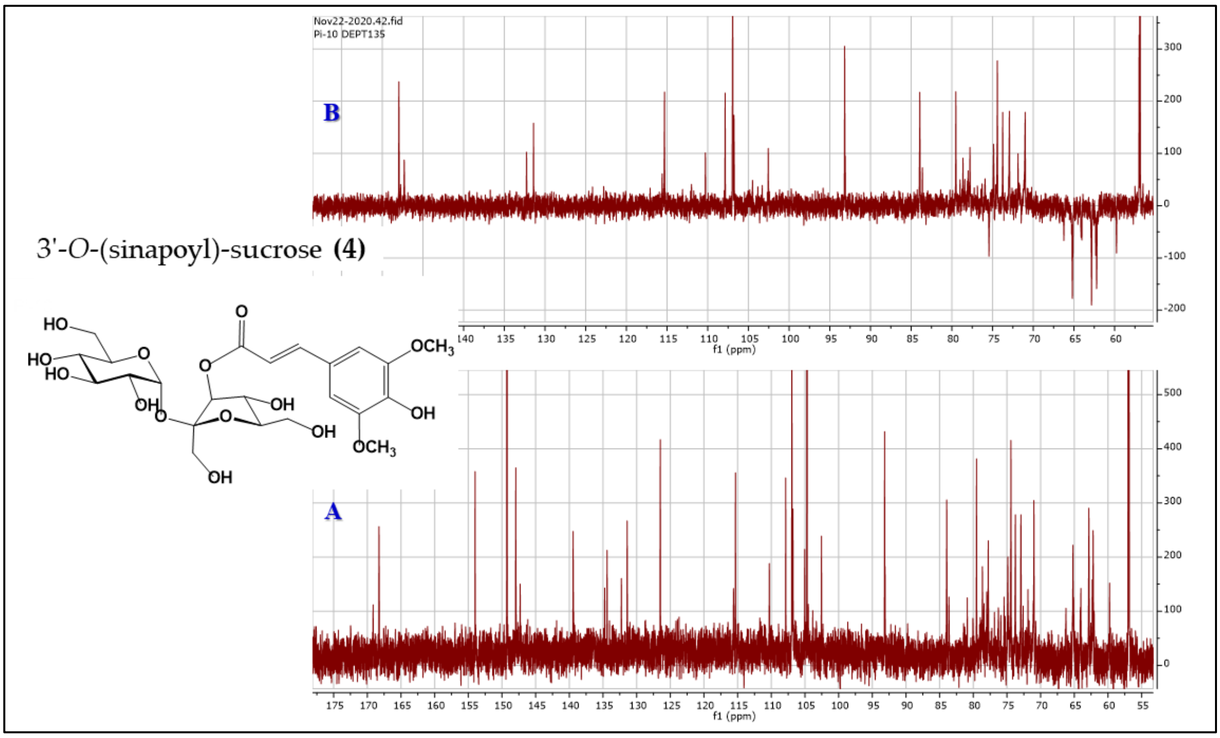

Figures S28&29. ^{13}C-NMR Spectrum (A) and DEPT-135 (B) of **3'-*O*-(sinapoyl)-sucrose (4)** (125 MHz, CD_3OD)

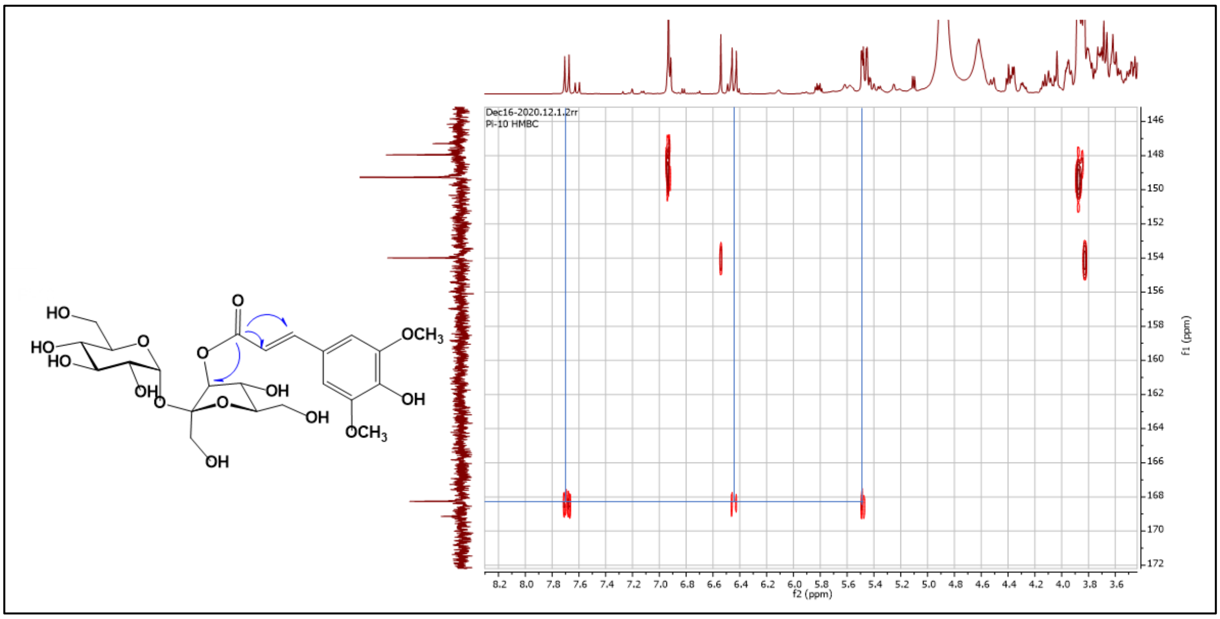

Figure S30. HMBC of **3'-*O*-(sinapoyl)-sucrose (4)**

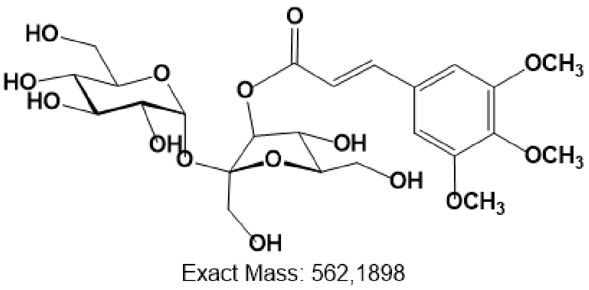

Figure S31. 3'-*O*-trimethoxy-cinnamoyl-sucrose (glomeratose) (5)

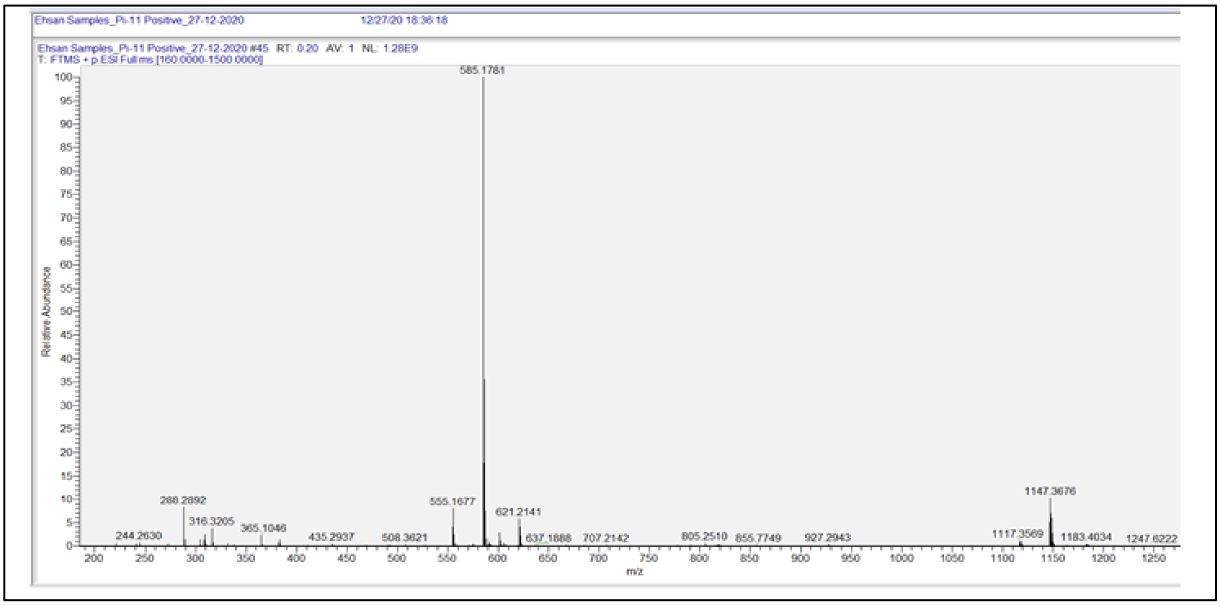

Figure S32. (+)-HRMS of **3'-*O*-trimethoxy-cinnamoyl-sucrose (glomeratose) (5)**

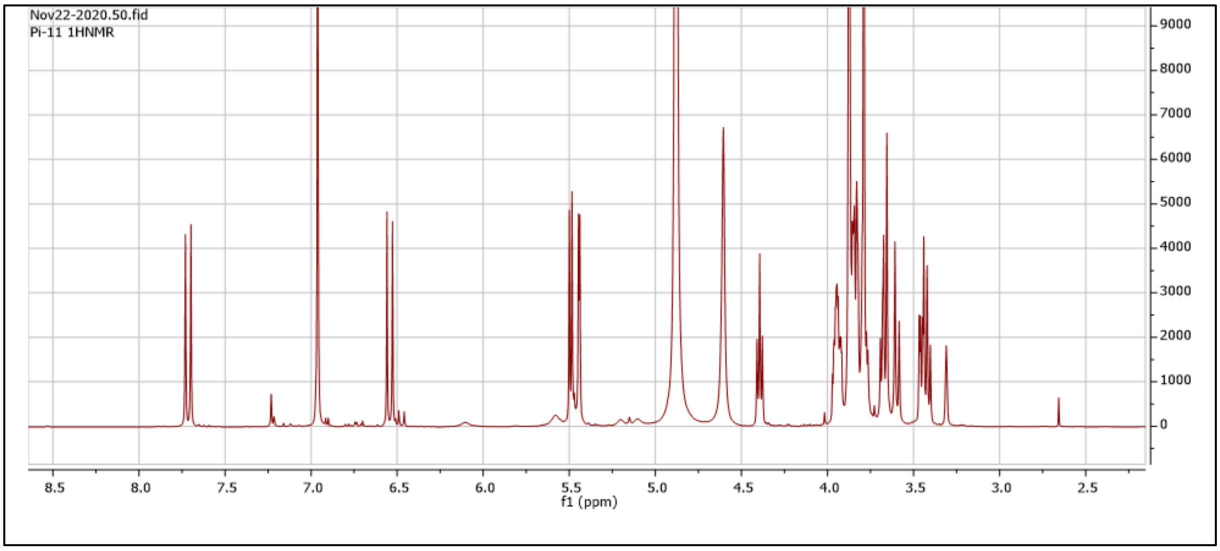

Figure S33. ^{1}H-NMR Spectrum of **3'-*O*-trimethoxy-cinnamoyl-sucrose (glomeratose) (5)** (500 MHz, CD_3OD)

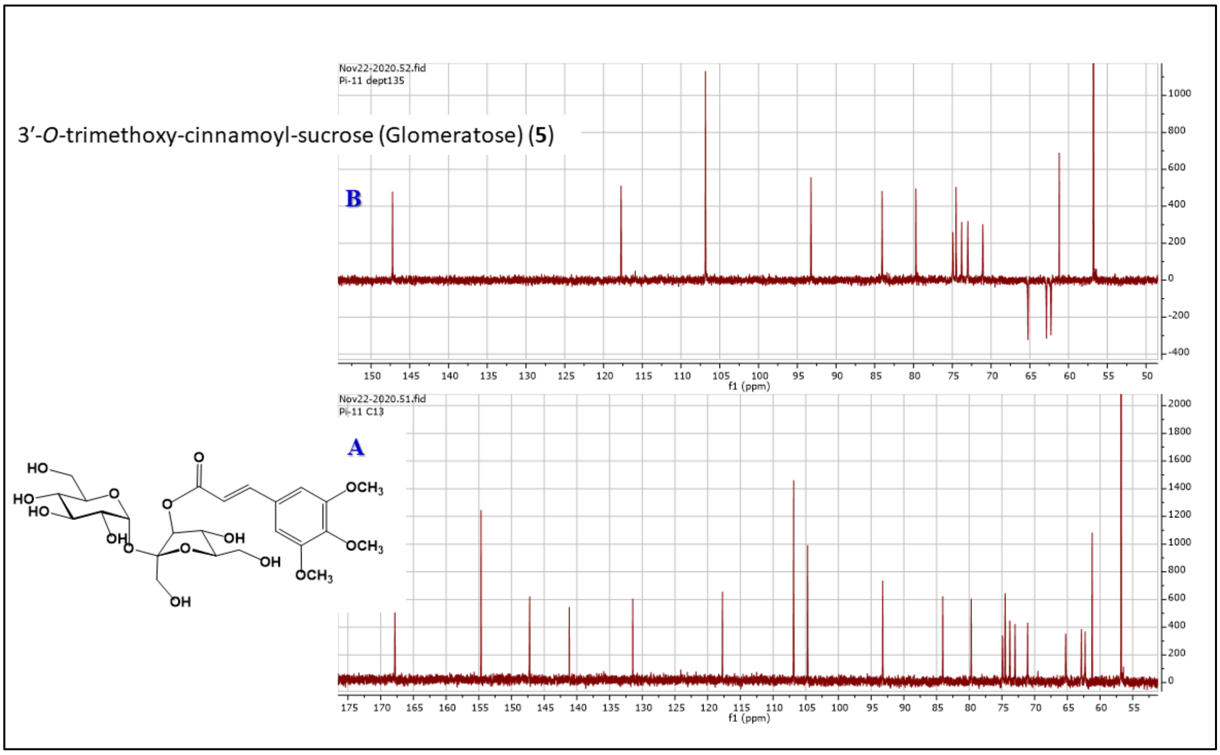

Figures S34&35. ^{13}C-NMR Spectrum (A) and DEPT-135 (B) of **3'-*O*-trimethoxy-cinnamoyl-sucrose (glomeratose) (5)** (125 MHz, CD_3OD)

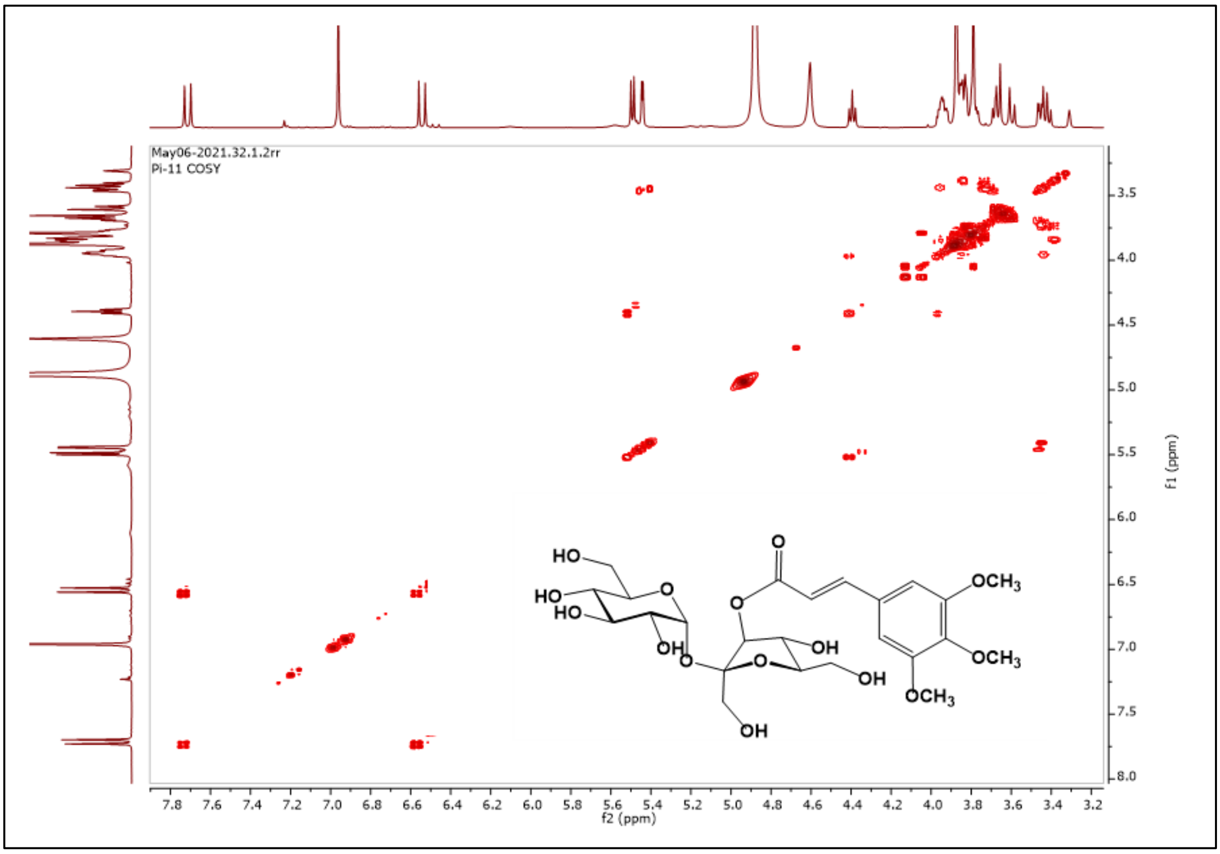

Figure S36. COSY of **3'-*O*-trimethoxy-cinnamoyl-sucrose (glomeratose) (5)**

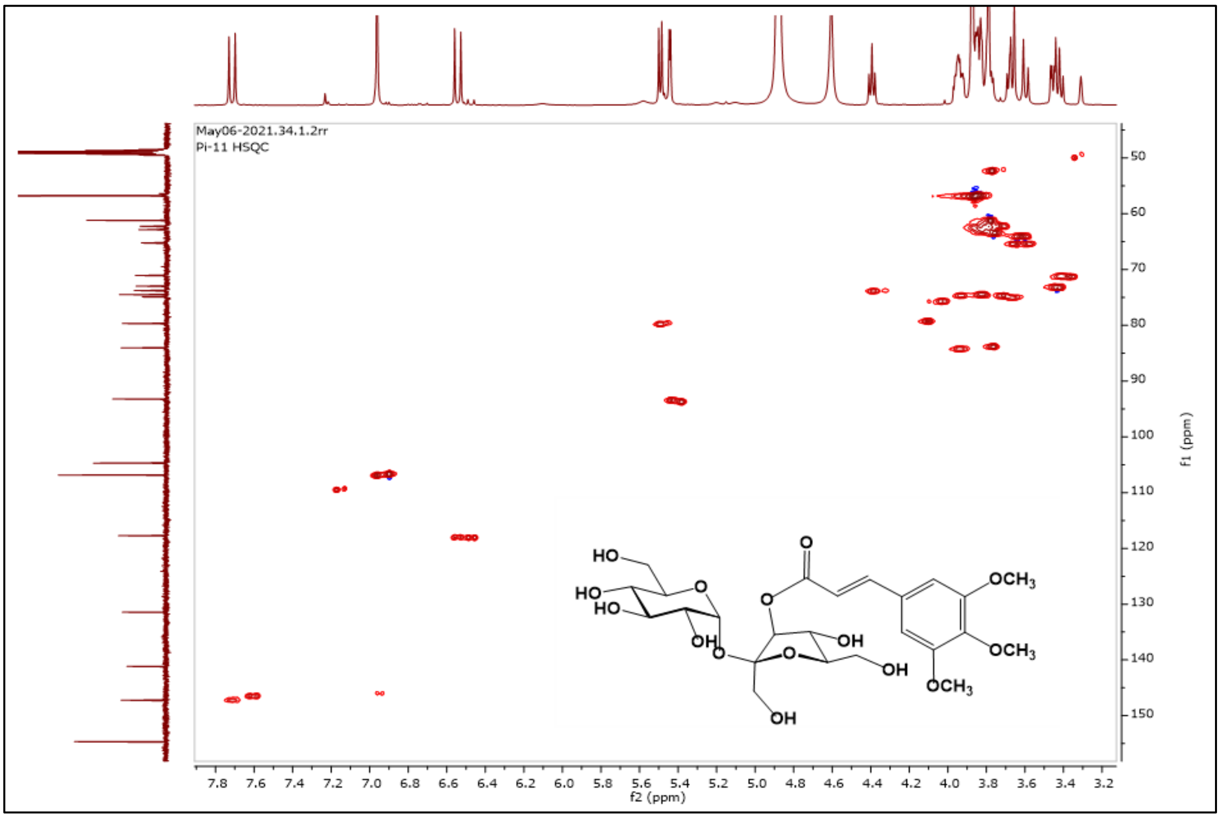

Figure S37. HSQC of **3'-*O*-trimethoxy-cinnamoyl-sucrose (glomeratose) (5)**

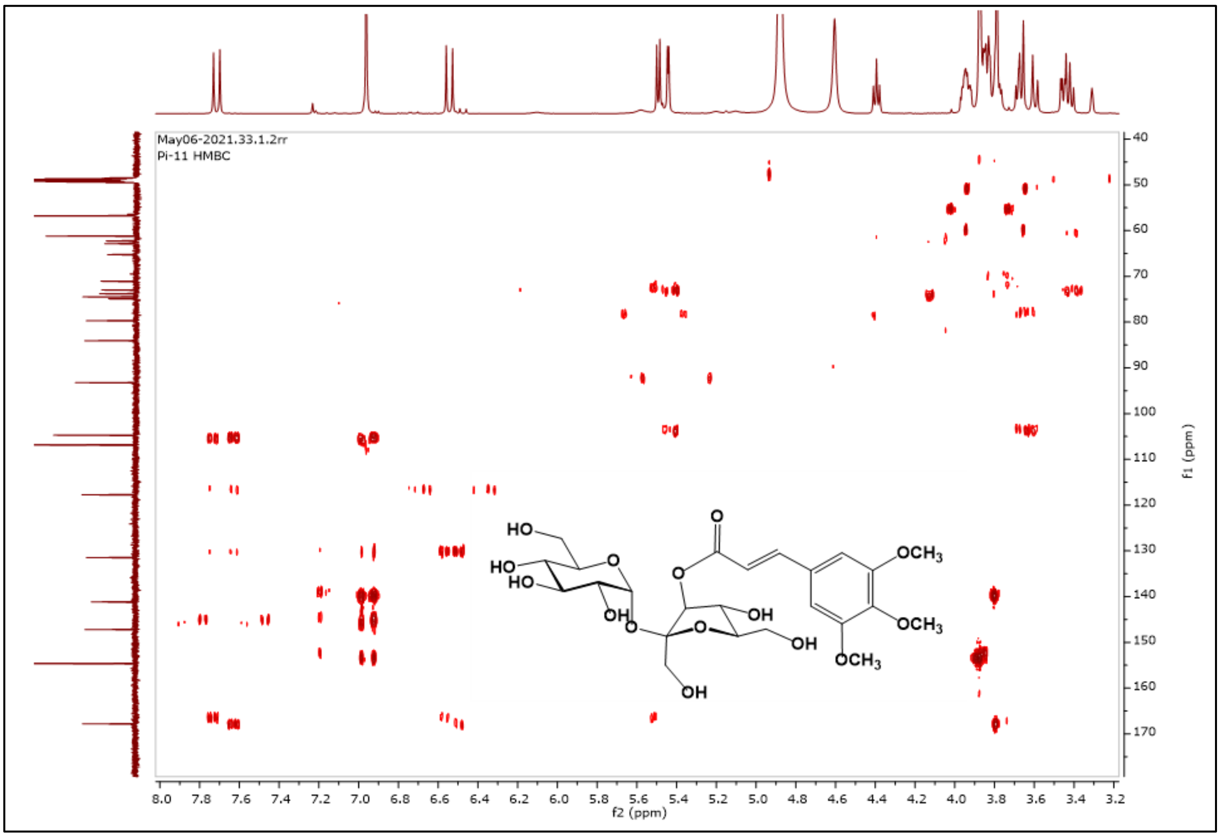

Figure S38. HMBC of **3'-*O*-trimethoxy-cinnamoyl-sucrose (glomeratose) (5)**

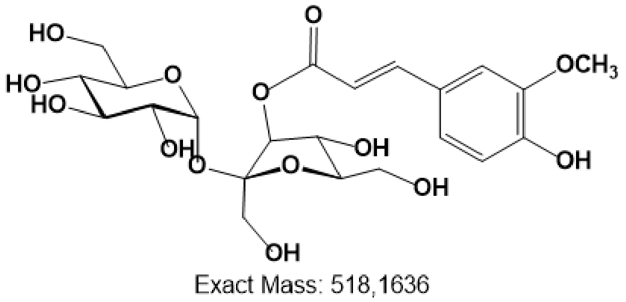

Figure S39. 3'-*O*-feruloyl-sucrose (sibiricose A5) (6)

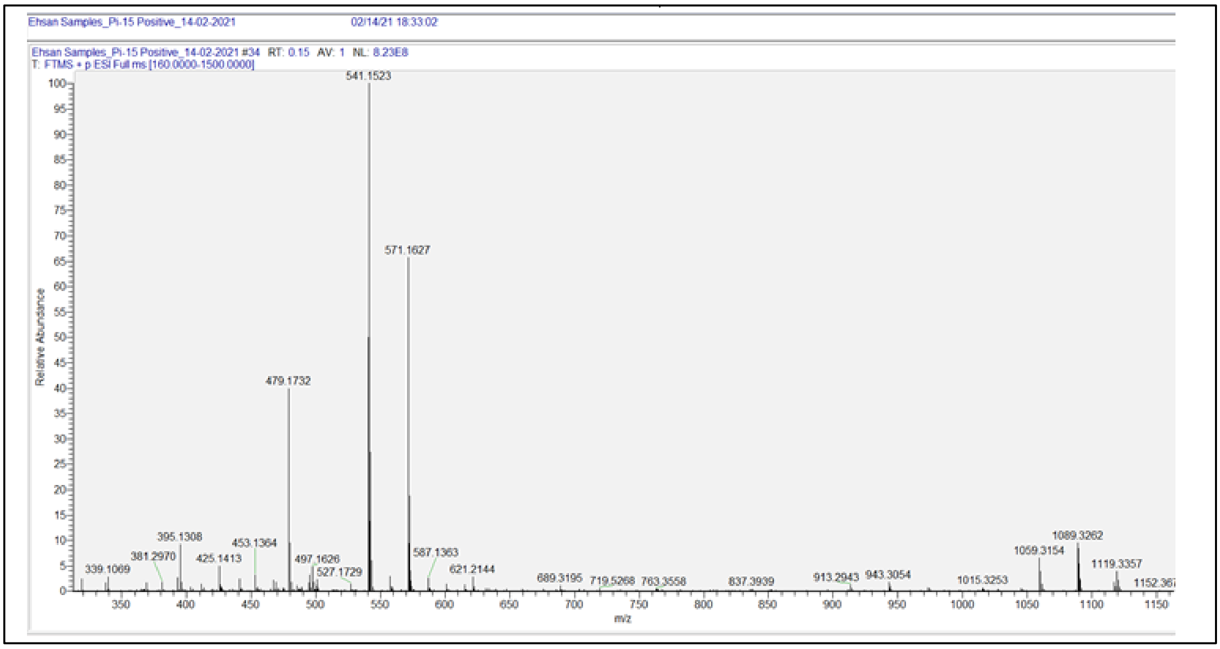

Figure S40. (+)-HRMS of **3'-*O*-feruloyl-sucrose (sibiricose A5) (6)**

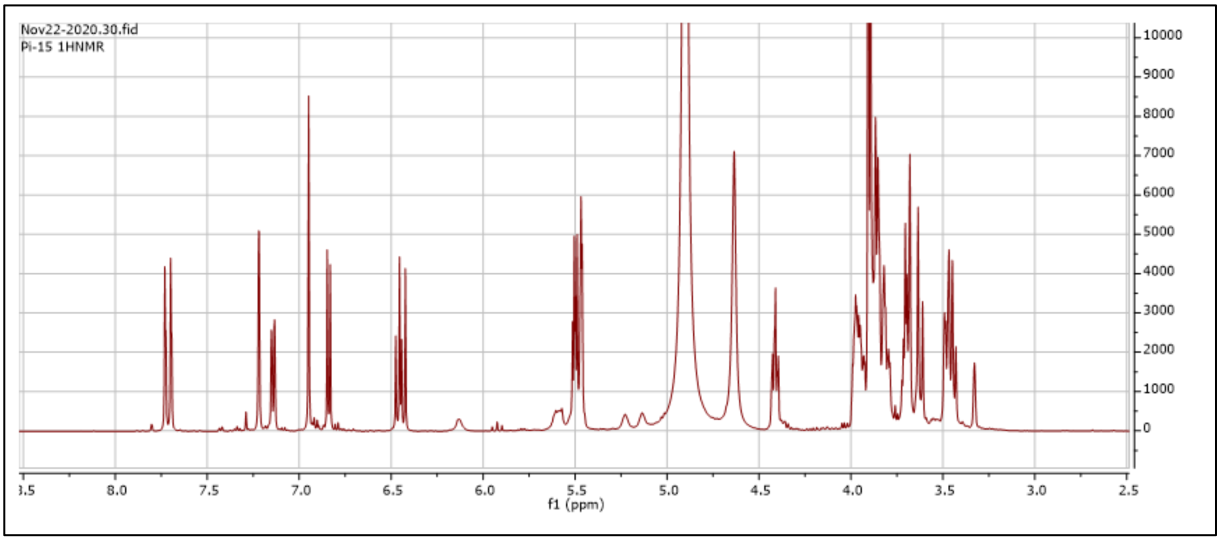

Figure S41. ^{1}H-NMR spectrum of **3'-*O*-feruloyl-sucrose (sibiricose A5) (6)** (500 MHz, CD_3OD)

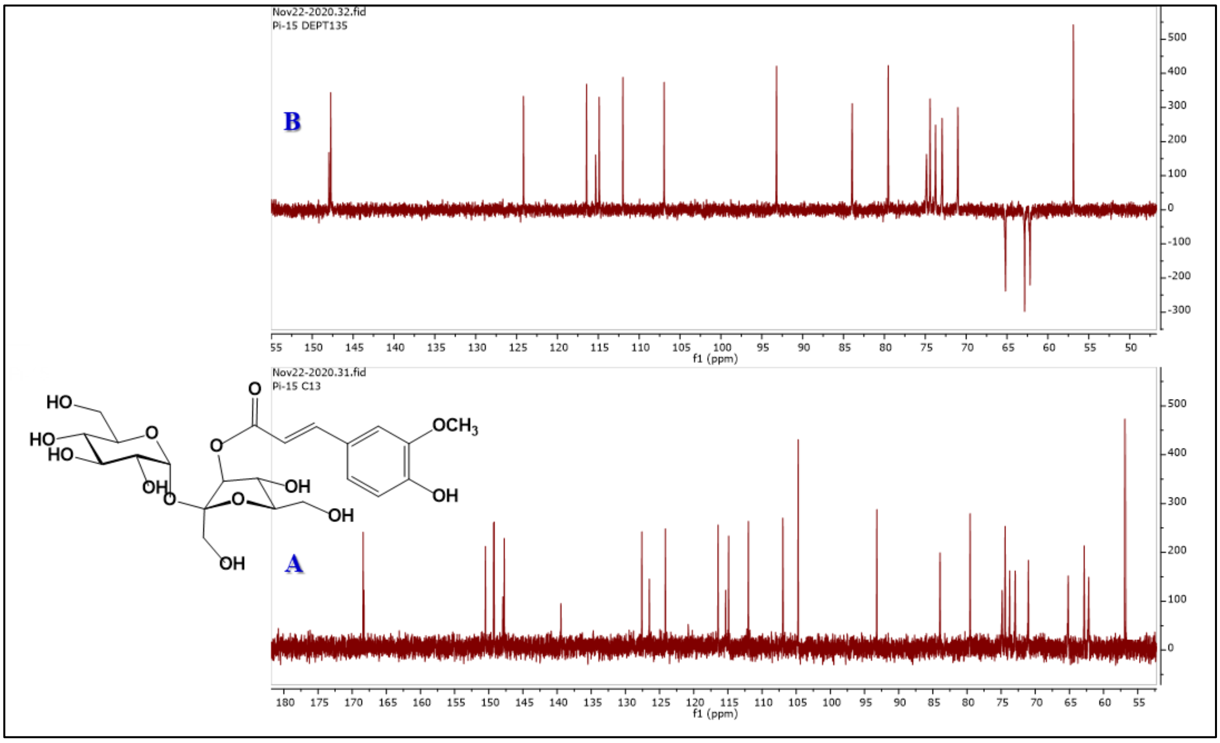

Figures S42&43. ^{13}C-NMR spectrum (A) and DEPT-135 (B) of **3'-*O*-feruloyl-sucrose (sibiricose A5) (6)** (125 MHz, CD_3OD)

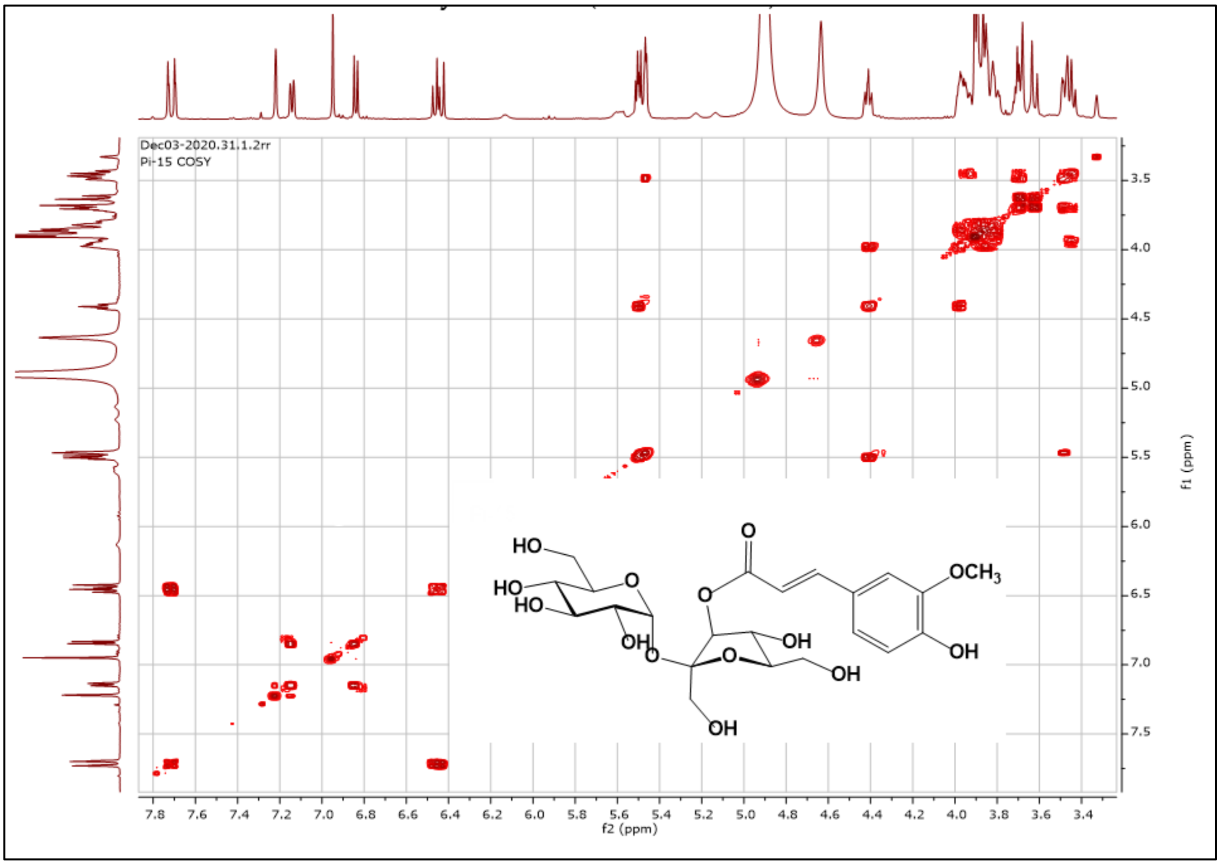

Figure S44. COSY of **3'-*O*-feruloyl-sucrose (sibiricose A5) (6)**

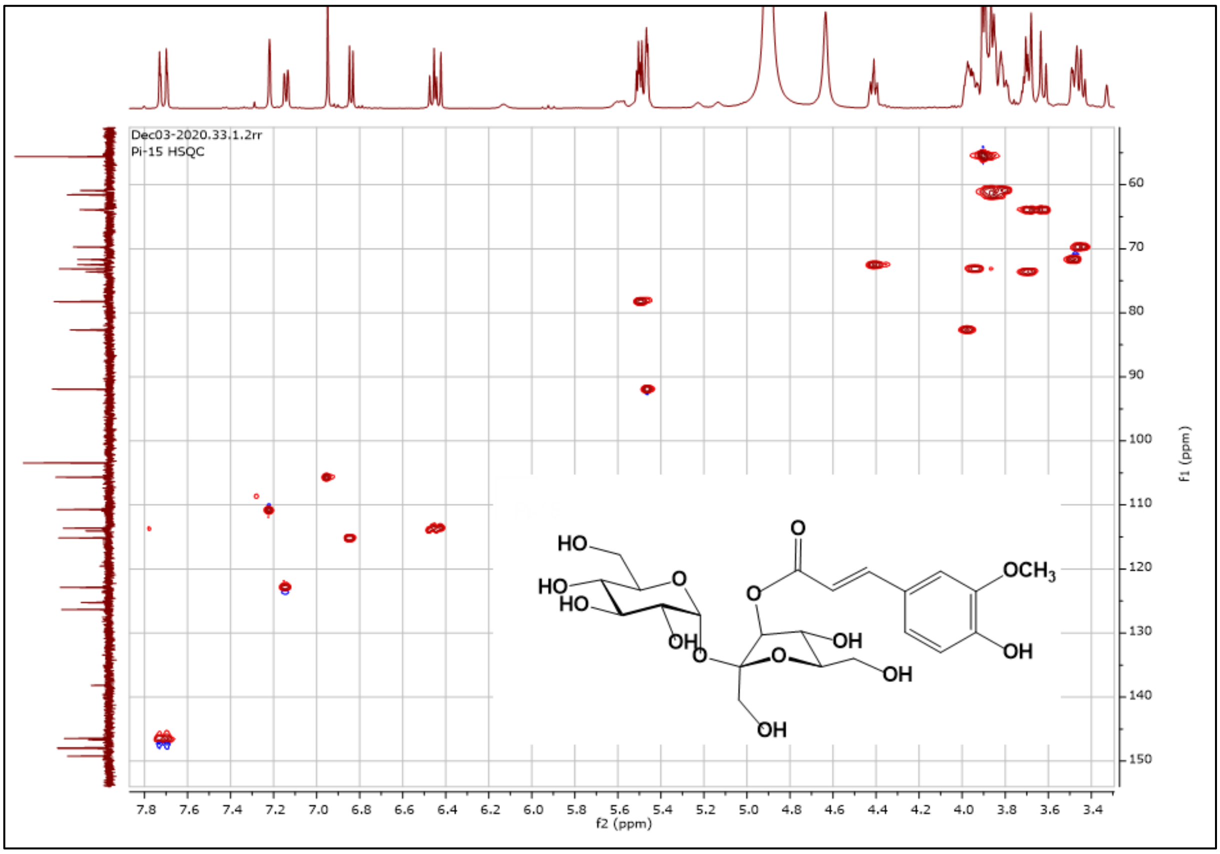

Figure S45. HSQC of **3'-*O*-feruloyl-sucrose (sibiricose A5) (6)**

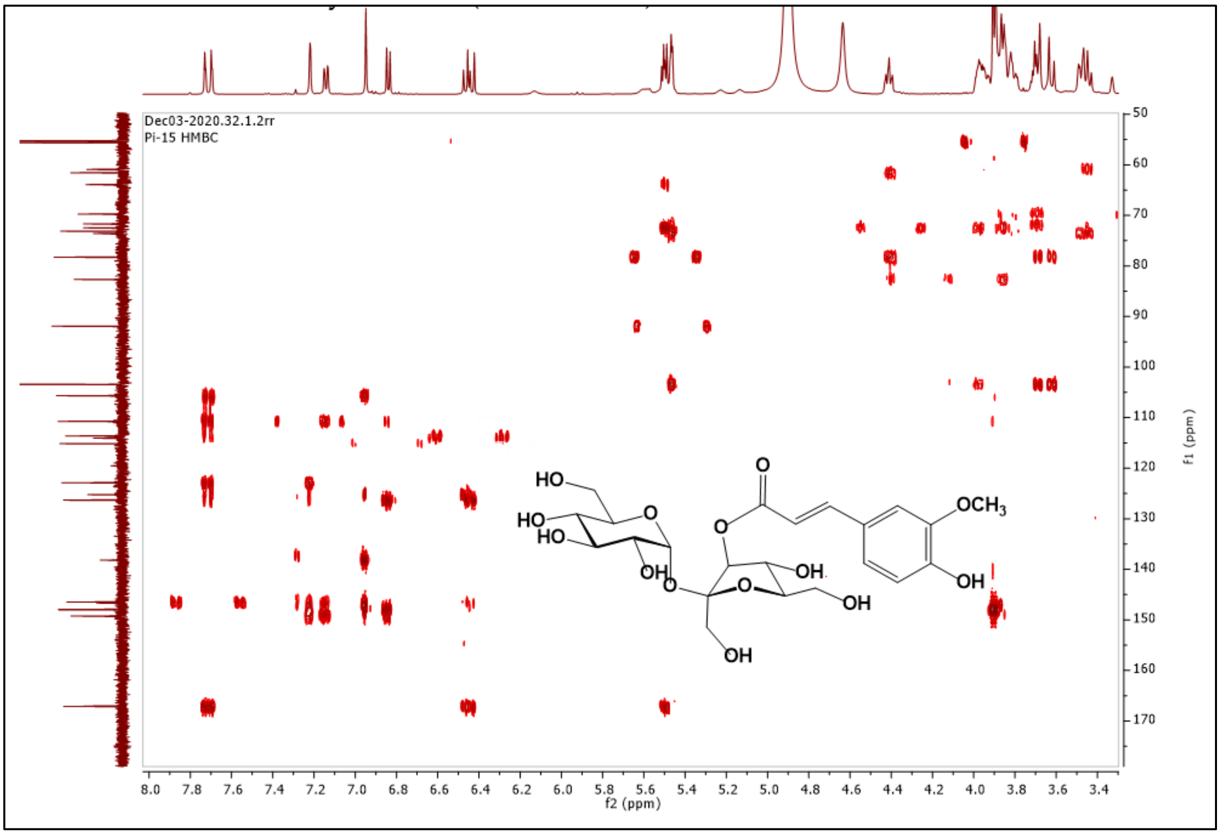

Figure S46. HMBC of **3'-*O*-feruloyl-sucrose (sibiricose A5) (6)**

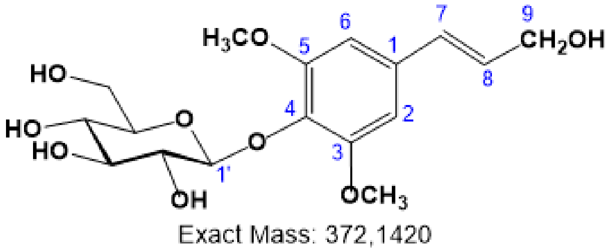

Figure S47. sinapyl alcohol 4-*O*-glucoside (syringin or eleutheroside B) (7)

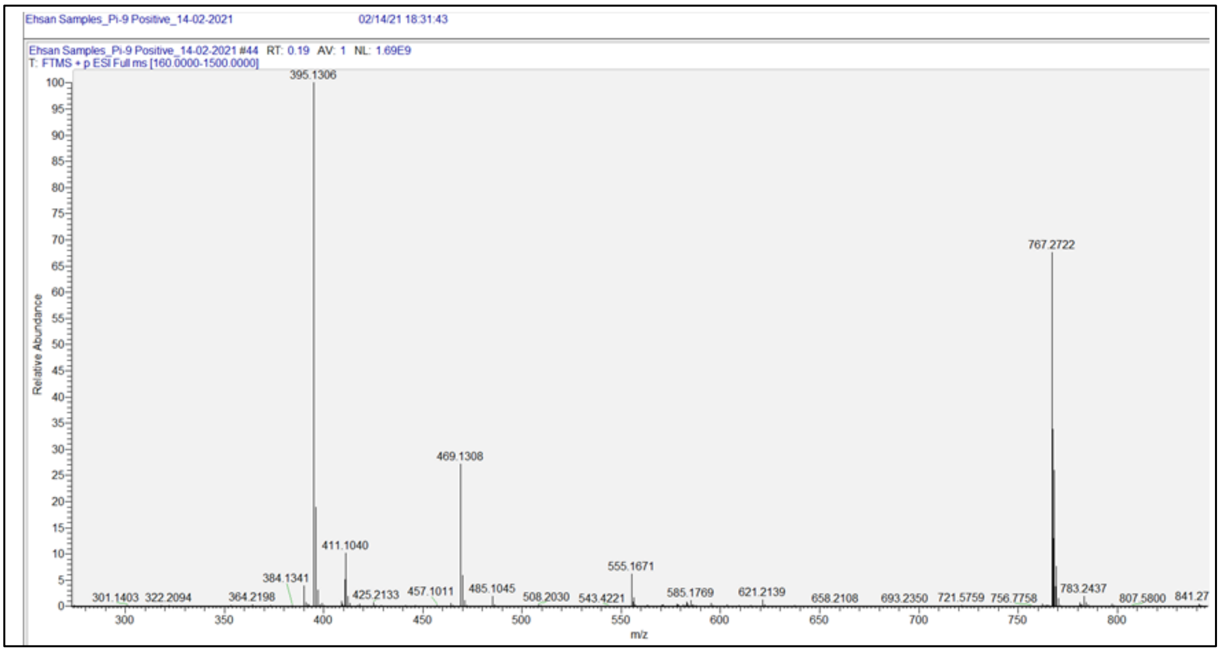

Figure S48. (+)-HRMS of **sinapyl alcohol 4-*O*-glucoside (syringin or eleutheroside B) (7)**

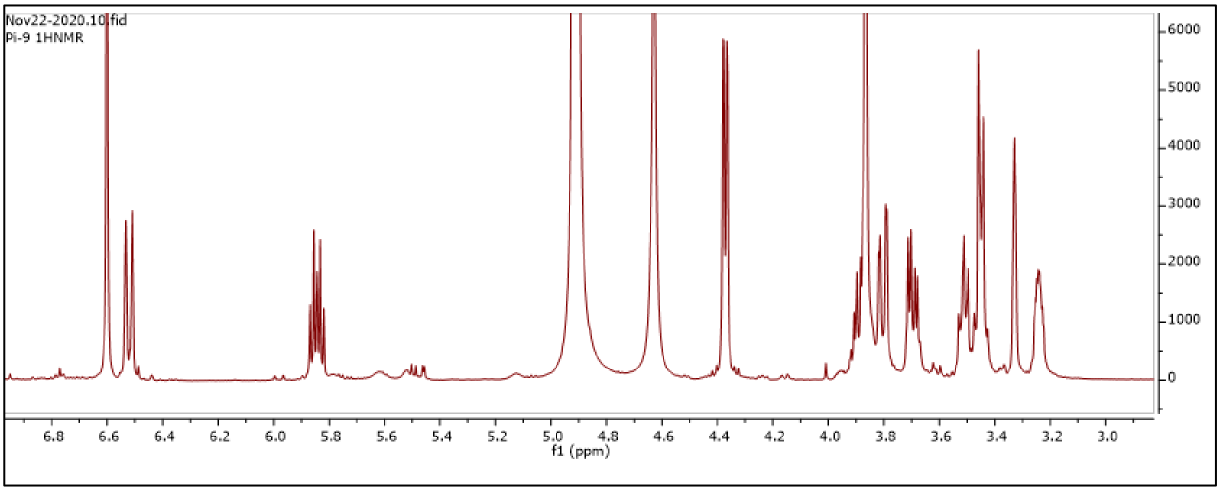

Figure S49. ^{1}H-NMR Spectrum of **sinapyl alcohol 4-*O*-glucoside (syringin or eleutheroside B) (7)** (500 MHz, CD_3OD)

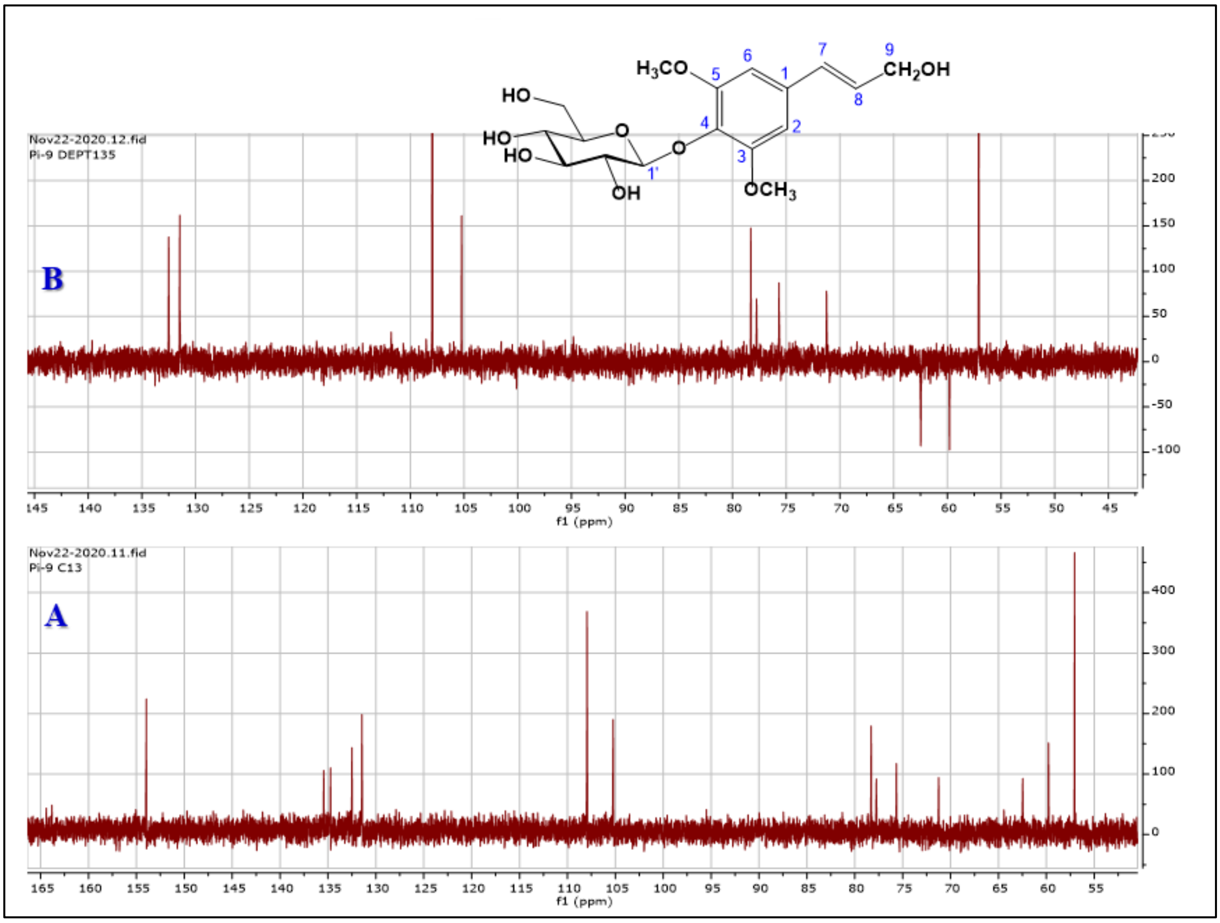

Figures S50&51. ^{13}C-NMR Spectrum (A) and DEPT-135 (B) of **sinapyl alcohol 4-*O*-glucoside (syringin or eleutheroside B) (7)** (125 MHz, CD_3OD)

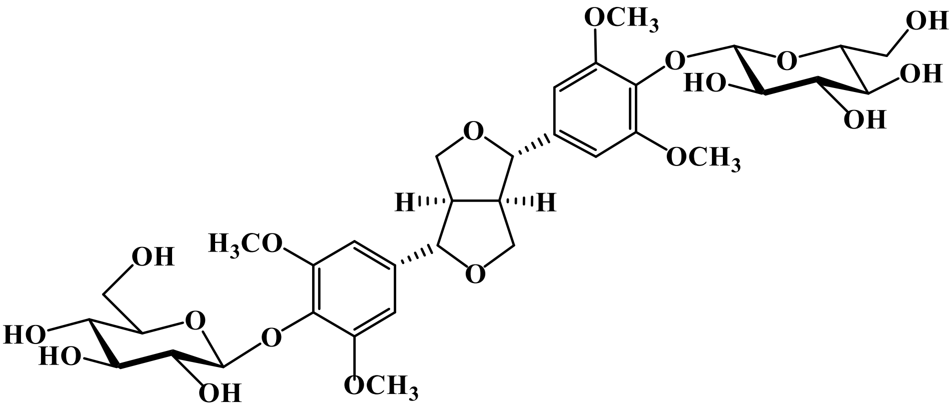

Figure S52. liriodendrin (8)

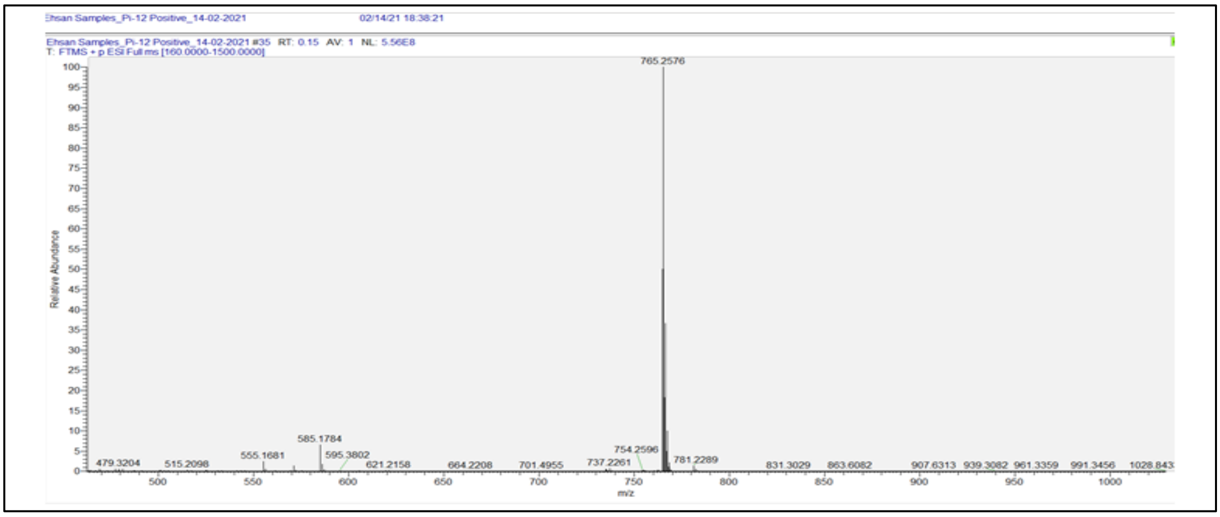

Figure S53. (+)-HRMS of **liriodendrin (8)**

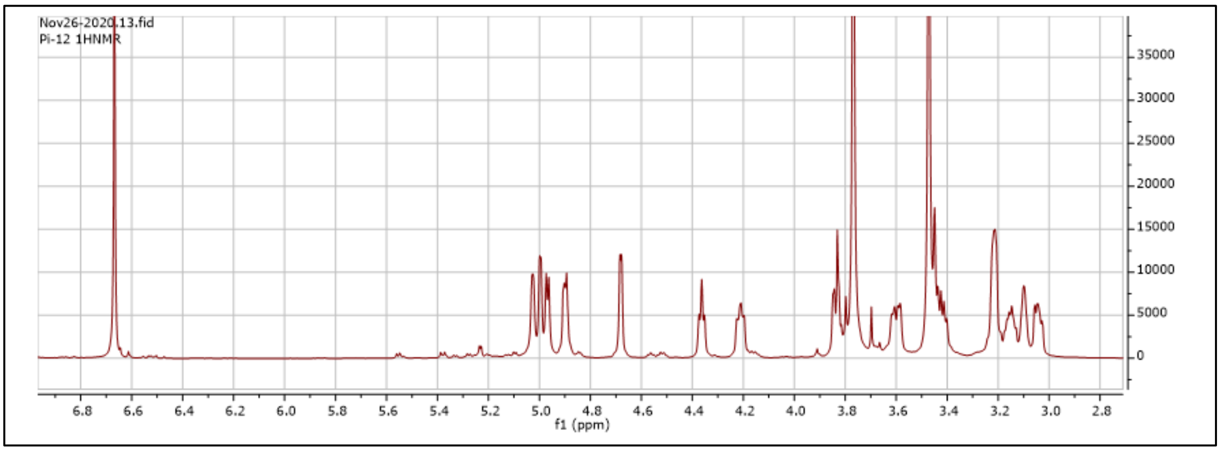

Figure S54. ^{1}H-NMR spectrum of **liriodendrin (8)** (500 MHz, CD_3OD)

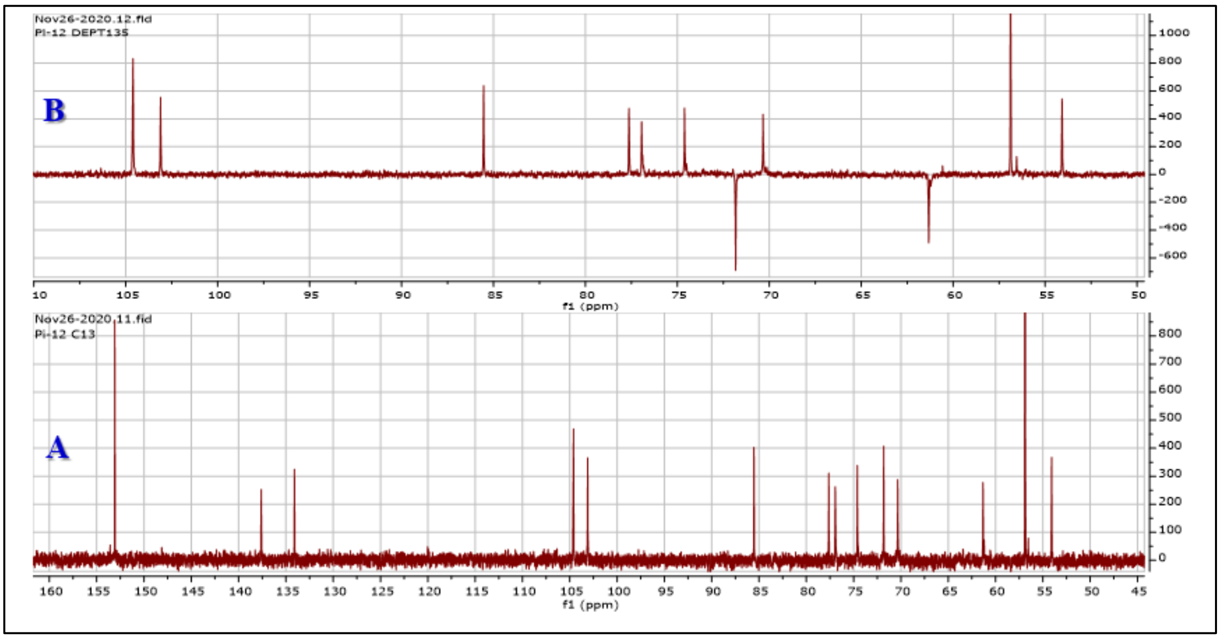

Figures S55&56. ^{13}C-NMR spectrum of **liriodendrin (8)** (125 MHz, CD_3OD)

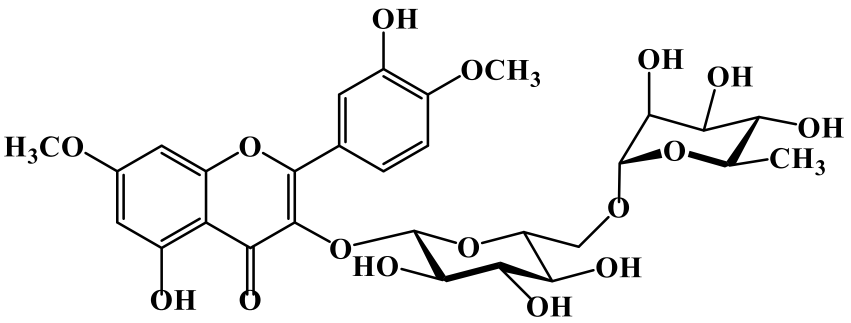

Exact Mass: 638,1847

Figure S57. 7,4'-di-*O*-methylquercetin-3-*O*-β-rutinoside (ombuin 3-*O*-rutinoside or ombuoside) (9)

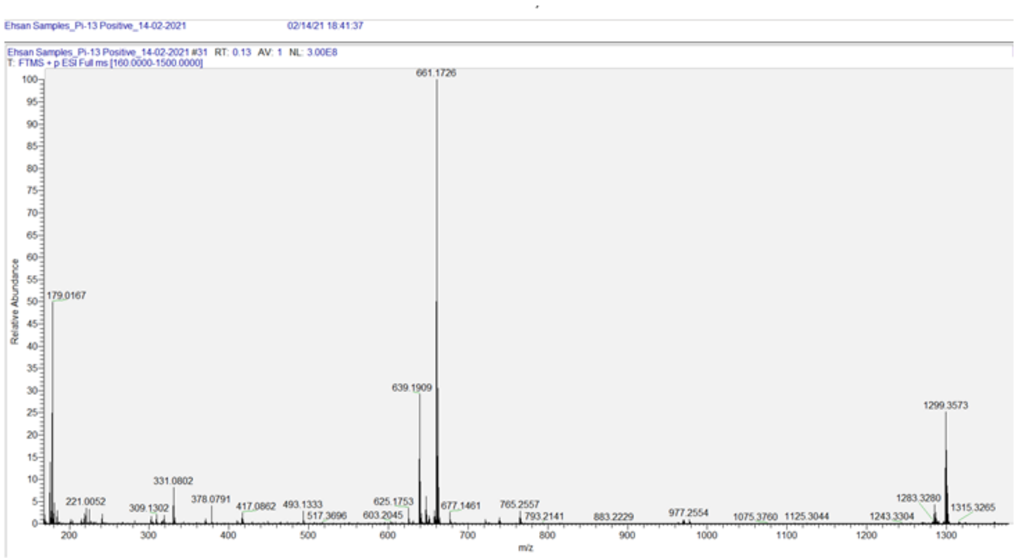

Figure S58. (+)-HRMS of **7,4'-di-*O*-methylquercetin-3-*O*-β-rutinoside (ombuin 3-*O*-rutinoside or ombuoside) (9)**

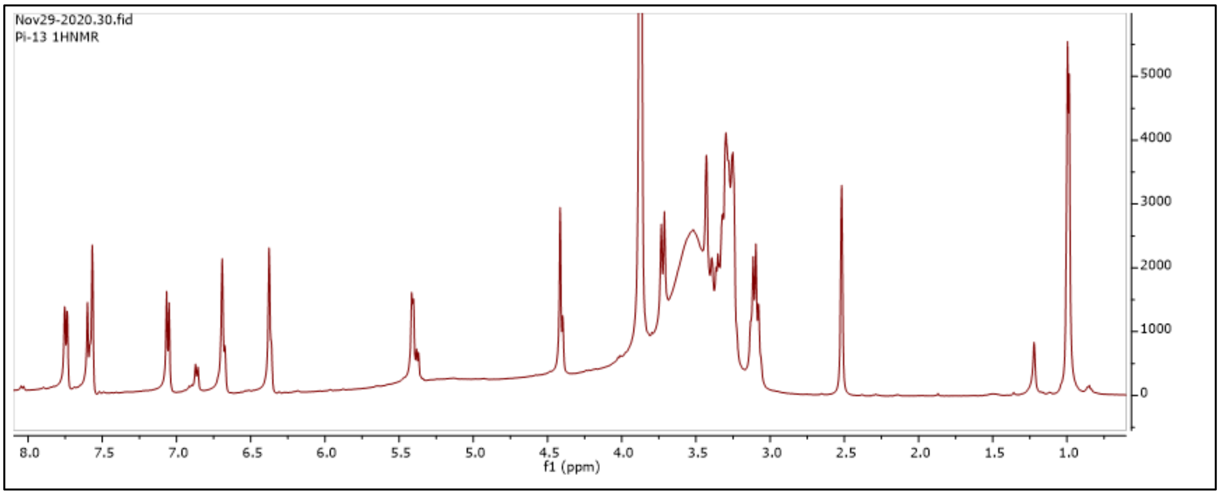

Figure S59. ^{1}H-NMR Spectrum of **7,4'-di-*O*-methylquercetin-3-*O*-β-rutinoside (ombuin 3-*O*-rutinoside or ombuoside) (9)** (500 MHz, CD_3OD)

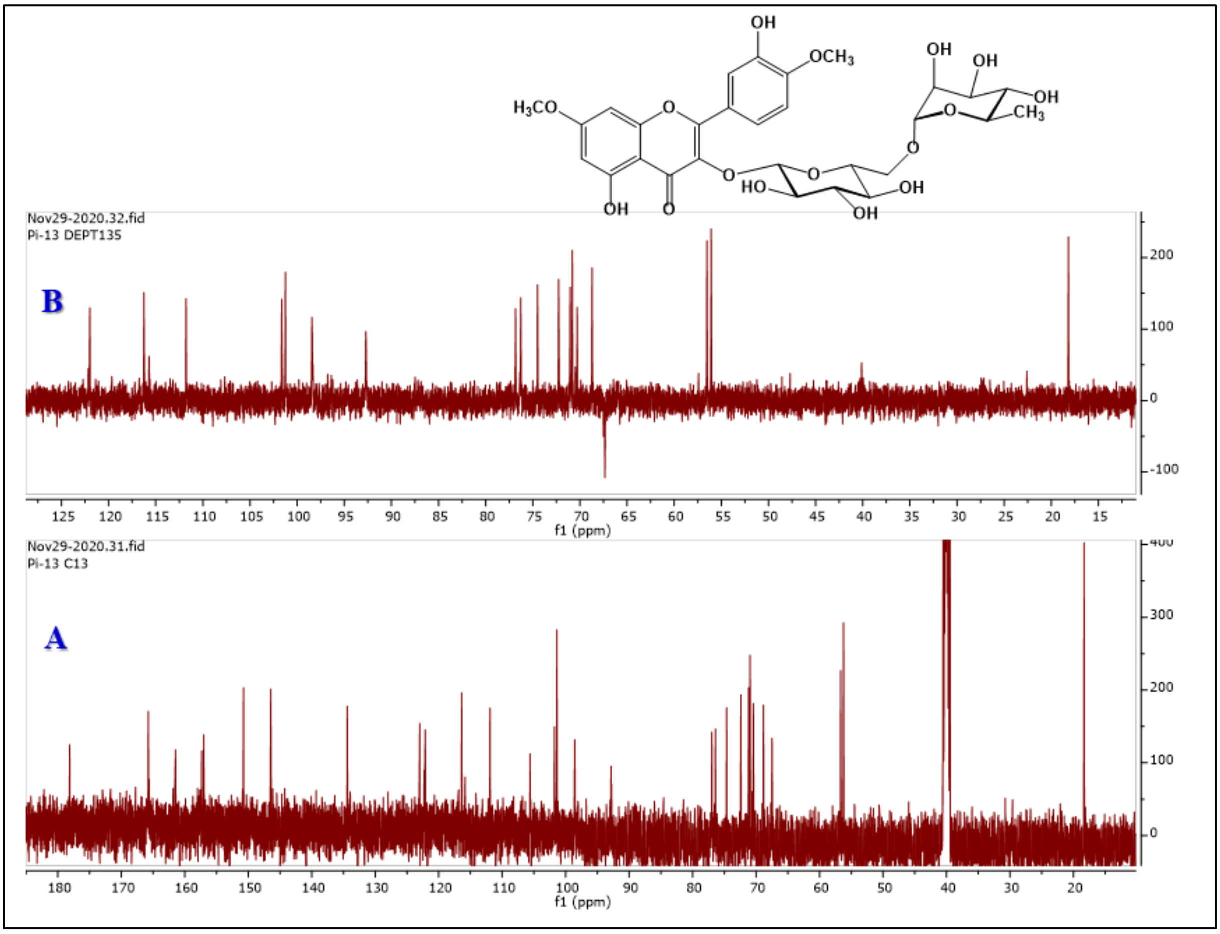

Figures S60&61. ^{13}C-NMR Spectrum (A) and DEPT-135 (B) of **7,4'-di-*O*-methylquercetin-3-*O*-β-rutinoside (ombuin 3-*O*-rutinoside or ombuoside) (9)**
